# Chart-Recorded Hourly Patient Fluid-Removal Intensity and 90-Day Mortality After a 12-Hour Landmark During Continuous Renal Replacement Therapy

**DOI:** 10.3390/medicina62101913

**Published:** 2026-10-03

**Authors:** Hasan Burak Toprak, Gürhan Taşkın, Hüseyin Levent Yamanel

**Affiliations:** Department of Intensive Care, Gülhane Training and Research Hospital, General Dr. Tevfik Sağlam Street No. 1, Keçiören 06010, Ankara, Türkiye; drgurhantaskin@gmail.com (G.T.); lyamanel@gmail.com (H.L.Y.)

**Keywords:** continuous renal replacement therapy, fluid removal, landmark analysis, mortality, restricted cubic spline, multiple imputation, MIMIC-IV

## Abstract

*Background and Objectives*: Appropriate fluid-removal intensity during continuous renal replacement therapy (CRRT) remains uncertain. We evaluated chart-recorded hourly patient fluid-removal intensity and mortality using a 12 h landmark. *Materials and Methods*: This retrospective MIMIC-IV v3.1 cohort included adults meeting recorded survival, hospital-observation, and CRRT criteria at the landmark. Exposure was the weight-normalized mean of retained hourly fluid-removal entries over 12 h; equivalence to delivered net ultrafiltration could not be validated. A four-knot restricted cubic-spline logistic model with 26 prespecified adjustment terms assessed 90-day mortality using 50 imputations for missing covariates. *Results*: Among 1453 patients, 847 (58.3%) had a recorded death within 90 days. Standardized risks were 57.6%, 57.5%, and 58.6% at the 25th percentile, median reference, and 75th percentile. Risk differences versus the reference were +0.1 percentage points (95% confidence interval, −2.4 to +2.6) and +1.1 percentage points (−2.6 to +4.9). Overall association (*p* = 0.475) and nonlinearity (*p* = 0.505) were not statistically detectable. Survival and complete-case confidence intervals spanned the null; complete-case uncertainty was greater. *Conclusions*: Early chart-recorded fluid-removal intensity was not detectably associated with 90-day mortality in this landmark cohort. Intervals remained compatible with clinically important differences; the findings do not define a delivered net-ultrafiltration target.

## 1. Introduction

Fluid accumulation is common in critically ill patients receiving continuous renal replacement therapy (CRRT) and is consistently associated with adverse outcomes [1,2,3]. Active fluid removal is therefore clinically consequential: insufficient removal may prolong congestion, whereas overly rapid removal may contribute to hemodynamic instability and organ hypoperfusion. Observational analyses of net ultrafiltration-related measures [4,5,6,7,8,9] and a recent meta-analysis [10] have reported heterogeneous associations with mortality across exposure definitions, time windows, case mix, and analytic strategies.

Two design issues are particularly important. First, fluid management during CRRT is dynamic: prescribed machine removal, delivered net ultrafiltration, and achieved patient fluid balance can diverge because of interruptions, concurrent inputs, and hemodynamic adaptation [11,12,13,14,15]. Accordingly, an electronic health record field may represent a charted setting or entry rather than achieved net fluid loss; the construct must be named and interpreted according to what was actually recorded. Second, classifying an exposure over an interval while beginning outcome follow-up before that interval is complete can create immortal-time bias. A landmark design aligns eligibility and follow-up after the exposure window [16,17].

Previous studies have used categorical and continuous exposure models, including splines, and differing early-treatment windows [4,5,6,7,8,9]. These approaches address related but nonidentical clinical questions. We evaluated a specifically defined MIMIC-IV chart field during the first 12 h using a prespecified continuous restricted cubic spline and a common landmark for eligibility and follow-up. We hypothesized that mortality might vary nonlinearly across the observed range, while recognizing that the analysis is associational rather than causal.

Our objective was to estimate the adjusted association between chart-recorded hourly patient fluid-removal intensity during the first 12 h of CRRT and all-cause mortality during the 90 days after a 12 h landmark. Secondary objectives examined survival time, alternative exposure definitions and coverage rules, 28-day mortality, in-hospital mortality, and exploratory modification by pre-CRRT fluid balance.

## 2. Materials and Methods

### 2.1. Study Design and Data Source

We conducted a retrospective cohort study using the Medical Information Mart for Intensive Care (MIMIC)-IV v3.1, a de-identified electronic health record database from Beth Israel Deaconess Medical Center that includes admissions from 2008 through 2022 [18,19,20]. The study followed a 12 h landmark design and is reported in accordance with STROBE and the RECORD extension [21,22]. The collection of patient information and creation of MIMIC-IV were reviewed by the Institutional Review Board at Beth Israel Deaconess Medical Center, which granted a waiver of individual informed consent and approved the data-sharing initiative [19]. The de-identified database was accessed through PhysioNet credentialing, and data use complied with the applicable data use agreement. Before journal submission, this retrospective secondary analysis underwent additional local review and approval by the University of Health Sciences, Gülhane Scientific Research Ethics Committee (Decision No. 2026-283; 31 August 2026). Patient-level MIMIC-IV data were accessed and analyzed exclusively by Hasan Burak Toprak, a credentialed PhysioNet investigator, within the controlled local analysis environment. Gürhan Taşkın and Hüseyin Levent Yamanel did not access restricted patient-level data; they reviewed the protocol, analysis logic, non-patient-level aggregate outputs, tables, figures, interpretation, and manuscript.

### 2.2. Cohort and Landmark Eligibility

**Figure 1:**
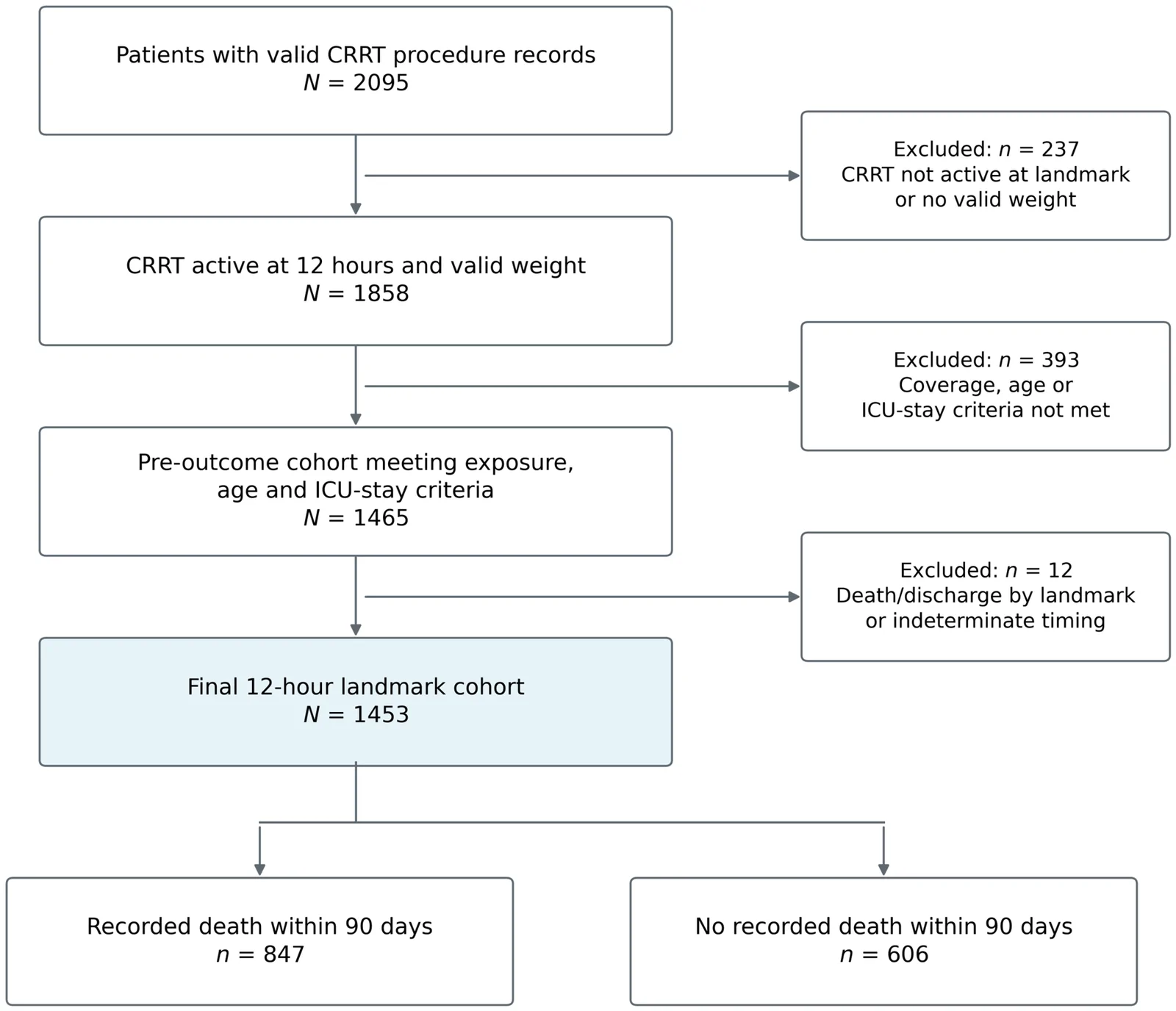
Patient-level cohort derivation. The final combined exclusion comprises deaths at or before the landmark, recorded discharge at or before the landmark, and temporally indeterminate boundary deaths, according to the timestamp-based algorithm. “No recorded death within 90 days” denotes the database endpoint rather than independently verified vital status. Descriptors of the selection stages are given in Table S1.

The analytic unit was the patient. The first valid CRRT procedure start per patient defined t0 and the index intensive care unit (ICU) stay; valid procedure rows required non-missing start and end times with end later than start. Overlapping or touching CRRT intervals within that stay were combined, without bridging gaps. Later ICU stays did not supply exposure records. Patients had to be aged at least 18 years, have t0 within the recorded ICU stay, and have a CRRT interval active at L = t0 + 12 h. Primary exposure coverage required at least 10 represented t0-anchored hourly bins, including at least four in each 6 h half, and valid body weight. Landmark eligibility was implemented from recorded timestamps: deaths at or before L, discharge at or before L, and date-only boundary deaths that could not be ordered relative to L were excluded according to the outcome algorithm (Supplementary Materials). The pre-outcome cohort comprised 1465 patients and the final cohort 1453 (Figure 1). No a priori sample-size calculation was performed; all qualifying patients were included.

### 2.3. Exposure

The exposure was chart-recorded hourly patient fluid-removal intensity during [t0, L), expressed in mL/kg/h. MIMIC-IV item 224191 is labeled “Hourly Patient Fluid Removal” (abbreviation, “Hourly PFR”), a numeric chartevents field in the Dialysis category recorded in mL. We retained numeric records from the index ICU stay during active CRRT intervals. At identical chart times, the latest stored record was selected; conflicting values tied at the latest storage time were excluded. The arithmetic mean of retained entries was divided by analytic weight. Thus, this was a record-weighted summary: distinct entries within an hour could contribute separately. It was not cumulative volume divided by elapsed treatment time, and unrecorded hours were not assigned zero. Negative and warning-flagged entries were retained in the primary specification and addressed in prespecified sensitivities. Negative entries may reflect documentation sign conventions, charting corrections, or errors; their specific meaning could not be verified. They were retained under the prespecified chart-field definition because a negative sign alone did not establish that a record was erroneous; sensitivity analysis F excluded negative entries (Table S2). Valid weight was 20–300 kg, selected in order from the index procedure, the closest daily weight before t0 within seven days, or the closest admission-weight entry before t0 in the index stay. All primary-cohort patients used procedure weight.

The field label and recording pattern are compatible with a device-level patient-removal measure, distinct from the patient’s overall fluid balance. We could not establish whether an entry represented a setting or an achieved volume or independently validate equivalence to delivered net ultrafiltration (UFNET). To describe its recording context, revision-stage comparisons examined items 225183 (“Current Goal”) and 226457 (“Ultrafiltrate Output”) in the same window. Comparator summaries were calculated within patients before summarizing across patients. Neither comparator had a documented accumulation interval in the inspected dictionary or concept definitions; numeric agreement coefficients were therefore not calculated (Table S3). These were comparisons of charted fields, not independent device validation.

**Figure 2:**
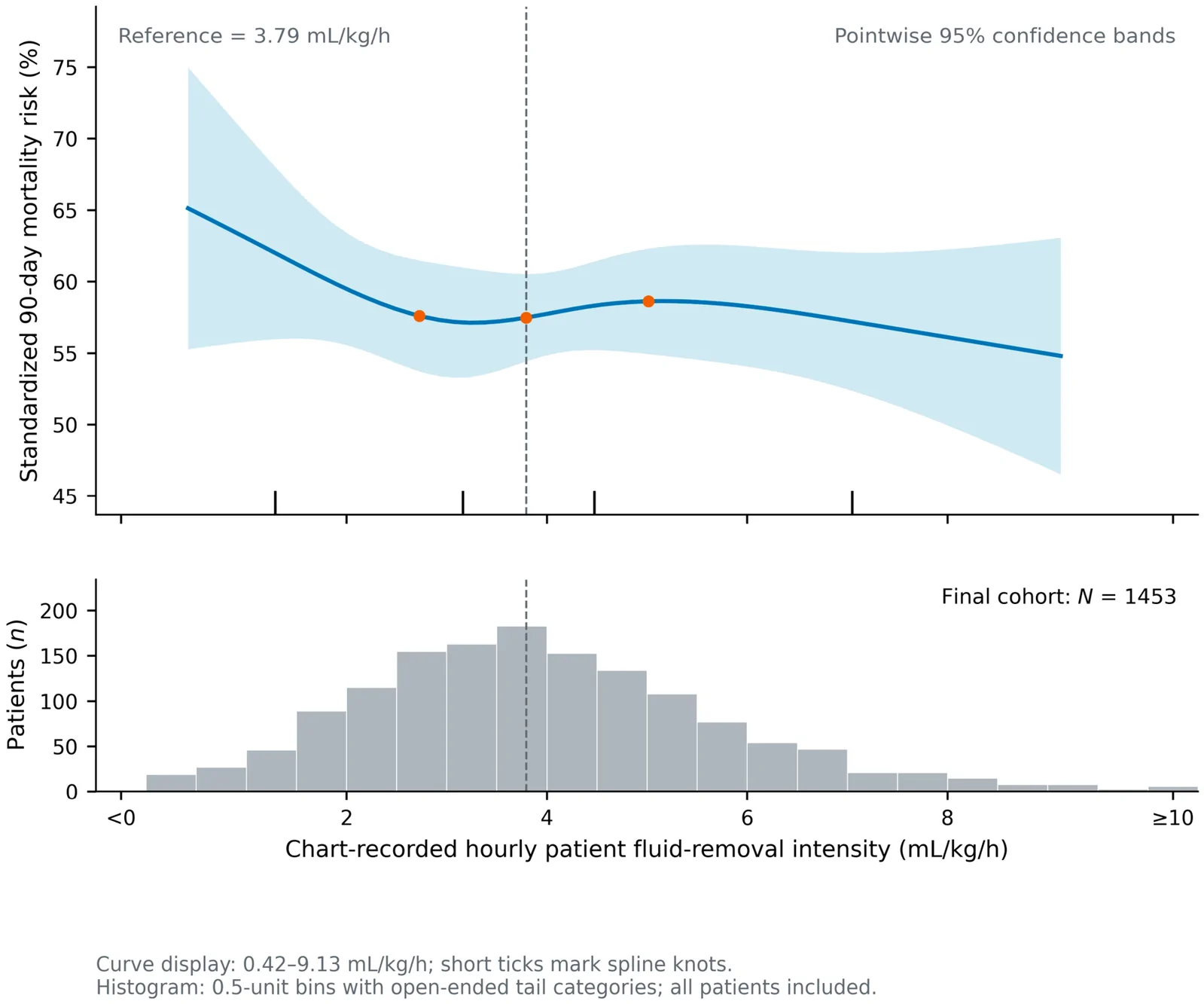
Standardized 90-day mortality across chart-recorded hourly patient fluid-removal intensity. The line and band show pooled spline estimates and pointwise 95% confidence intervals, using 50 imputations. The reference is 3.79 mL/kg/h, with Q25 = 2.73 and Q75 = 5.02 mL/kg/h. Orange points mark Q25, the reference, and Q75; the dashed line marks the reference. Short ticks mark the spline knots, fixed from the 1465-patient pre-outcome distribution. The curve uses the pre-outcome 1st–99th percentile range (0.42–9.13 mL/kg/h); the histogram displays all 1453 analytic-cohort patients, including outer categories. Observations outside the curve display range were retained in the model. The RD curve is provided in Figure S1.

Exposure was modeled continuously. The four spline-knot percentile locations (5th, 35th, 65th, and 95th) were prespecified; numerical knots of 1.29, 3.17, 4.48, and 7.05 mL/kg/h came from the 1465-patient pre-outcome distribution and remained fixed. The reference was 3.79 mL/kg/h, with contrasts at 2.73 (Q25) and 5.02 (Q75). The pre-outcome 1st–99th percentile display range was 0.42–9.13 mL/kg/h. Figure 2 uses this fixed range for the curve and displays the full analytic-cohort histogram, including tail categories; exact anchors are given in the Supplementary Materials.

### 2.4. Outcomes

The primary outcome was all-cause mortality within 90 days after L. Fixed-horizon death indicators used the patients.dod date-of-death field on or before the calendar date of L plus 90 days, or plus 28 days for the secondary endpoint. The hospital death timestamp did not override this date field for these binary endpoints; it was used for landmark ordering, the post-landmark in-hospital endpoint, and fractional survival times where applicable. The hospital-expire flag was a quality-control check, not a death time. MIMIC-IV combines hospital and state death records collected two years after the last discharge and censors death dates beyond one year after the final discharge [19]. The 90-day horizon therefore falls within its nominal observation window. Patients without a recorded death within the horizon were classified as having no recorded death, conditional on completeness of the linkage. This does not establish individually verified follow-up. Survival timing and day-90 administrative censoring are detailed in Table S4.

### 2.5. Covariates

The prespecified adjustment set contained 26 terms: age; sex; race; admission type; first ICU type; calendar period; ICU-admission-to-CRRT time; CRRT mode; Charlson comorbidity index [23]; chronic kidney disease; heart failure; chronic liver disease; malignancy; custom pre-CRRT Sequential Organ Failure Assessment (SOFA) total [24]; documented suspected infection before t0; mechanical ventilation and norepinephrine-equivalent dose at t0; median mean arterial pressure in the preceding 6 h; closest lactate and pH in the preceding 6 h; closest creatinine, blood urea nitrogen, bicarbonate, and potassium in the preceding 24 h; urine output over the preceding 6 h; and cumulative ICU-admission-to-t0 fluid balance. Urine output and fluid balance were weight-normalized. Race was included as recorded as a social, rather than biological, covariate representing potential differences in access, treatment, and structural factors. Source fields, windows, coding, adjustment rationale, and model degrees of freedom appear in Table S5.

Physiologic windows ended before t0 or described support active at t0. Charlson and the four comorbidity indicators instead came from administrative diagnoses for the whole index admission; their availability before t0 cannot be verified. The suspected-infection indicator used antibiotic–culture pairing with both records before t0 and did not require a contemporaneous SOFA change; it is not a validated sepsis diagnosis. The custom SOFA total summed six component scores using pre-t0 measurements and support status at t0. The renal component used creatinine; the cardiovascular component combined prior-24-h minimum MAP with norepinephrine-equivalent dose active at t0. The respiratory component used ventilation status at t0. Component-specific windows are detailed in Table S5. It should not be assumed identical to a fully observed standard SOFA assessment.

### 2.6. Missing Data

Missing covariates were handled by chained-equations multiple imputation (MI) with 50 imputations, 50 iterations, and seed 42 [25,26]. Continuous variables used predictive mean matching. Ordered SOFA components used proportional-odds models, except the cardiovascular component, which used multinomial regression; its observed score levels were 0, 1, 3, and 4. CRRT mode used binary logistic imputation. Total SOFA was reconstructed from the six completed component labels after imputation. Exposure and outcomes were not imputed; outcome and exposure information was included as predictors. Table S6 summarizes scenario-specific imputation; Tables S7–S9 and the accompanying predictor-matrix file document methods, predictors, missingness by outcome and exposure quartile, observed/imputed distributions, and convergence summaries. These diagnostics assess computation and distributions, not the missing-at-random assumption.

### 2.7. Statistical Analysis

The primary estimand was the adjusted association with mortality at a fixed 90-day horizon. Logistic regression was fitted in each completed dataset with a four-knot restricted cubic spline for exposure [27]. The 26 adjustment terms contributed 41 parameters; the three exposure spline parameters gave 44 degrees of freedom excluding the intercept. All continuous adjustment covariates were linear in the primary model. MI-compatible D1 multivariate Wald tests compared the full model with models omitting the exposure terms (overall association, 3 df) or retaining only linear exposure (nonlinearity, 2 df). Adjusted odds ratios (ORs) were pooled on the log-OR scale. Standardized mortality risks were calculated by g-computation at Q25, the reference, and Q75 over each analysis population; risk differences (RDs) were the corresponding differences from the reference. Within-imputation risk and RD variances used the delta method with the full coefficient covariance matrix. Rubin pooling was applied on the risk and RD scales, with t-based pointwise 95% intervals incorporating within- and between-imputation uncertainty.

Prespecified robustness analyses varied the exposure boundary, hourly coverage, within-bin summary, negative/warning-record handling, weight source, and exclusion of end-stage kidney disease or known slow continuous ultrafiltration (Table S2). Cox models used the same exposure spline and adjustment set, Efron ties, follow-up from L, and day-90 administrative censoring. Exposure-specific proportionality was assessed by a pooled joint test of three spline-by-log(time/28) interactions. Global proportional hazards were assessed with cox.zph using Kaplan–Meier-transformed time and term-level testing within each imputed dataset; these descriptive diagnostics were not pooled or used for exposure inference or model selection. Restricted mean survival time (RMST) provided a complementary survival summary [28]. We estimated adjusted contrasts by direct regression of restricted survival time on the same predictors, with heteroskedasticity-consistent (HC3) covariance and Rubin pooling. This construction treated recorded death times as events and other observations as 90 days, with no earlier censoring modeled. Secondary logistic analyses addressed 28-day and in-hospital mortality; modification by pre-t0 fluid balance was exploratory. The planned post-t0 hemodynamic sensitivity was not executed because its variables had not been specified in sufficient operational detail. *p*-values are nominal without multiplicity correction, and overlapping analyses are not independent replications.

The original internally dated analysis plan, its pre-outcome amendment log, and a retrospective implementation history are supplied with the data-free code package. Their timing is supported by internal provenance rather than external registration or an independent timestamp. The Supplementary Materials distinguish the restarted study, subsequent implementation changes, and work specified and executed during revision.

### 2.8. Revision-Stage Assessments

The existing imputation objects were reused to reproduce the primary estimates and describe apparent calibration, influence on exposure contrasts, and collinearity of the design matrix. Calibration was descriptive for an association model and was not externally or internally validated. A complete-case model retained the same exposure and covariate specification, using ordinary maximum likelihood when estimable; the planned fallback for separation was bias-reduced fitting, with non-estimability to be reported rather than selecting covariates from results. A separate sensitivity gave three-knot splines to age, lactate, norepinephrine-equivalent dose, pre-t0 cumulative fluid balance, and 6 h urine output, using observed 10th, 50th, and 90th percentiles fixed before those fits. The complete-case sensitivity was declared in the original plan and was reassessed and reported in detail during revision; the covariate-spline sensitivity was specified at revision. The reused imputations were not regenerated to incorporate the additional nonlinear covariate terms, which limits this functional-form assessment. Complete-case risks were standardized over the complete-case population and used delta-method Wald intervals; their association tests were likelihood-ratio tests. No new imputations were generated for these assessments (Tables S10 and S11).

### 2.9. Software and AI-Assisted Coding

Analyses were conducted in R version 4.5.1 using mice 3.19.0, rms 8.1-1, Hmisc 5.2-6, survival 3.8-3, MASS 7.3-65, and nnet 7.3-20.

Generative AI tools (Anthropic Claude Code and OpenAI ChatGPT, including Codex; models: Claude Opus 5, Claude Fable 5.1, GPT-5.6 Sol, and GPT-6 Astra, accessed in 2026) were used to assist with SQL/R code drafting and debugging, code review, language refinement, consistency checks, and manuscript formatting. All patient-level analyses were executed locally by the credentialed investigator; restricted patient-level MIMIC-IV data were not entered into or uploaded to hosted generative AI services. The authors reviewed and verified all AI-assisted outputs and take full responsibility for the content of the article.

## 3. Results

### 3.1. Cohort and Outcomes

Of 2095 patients with a valid index CRRT procedure, 1858 remained after the active-at-landmark and weight requirements; 1465 met pre-outcome age, ICU-timing, and coverage criteria, and 1453 met the final timestamp-based landmark criteria (Figure 1). The final analysis retained 1453 of 2095 patients (69.4%). Within 90 days after L, 847 (58.3%) had a recorded death and 606 had no recorded death under the endpoint algorithm. There were 719 deaths by day 28 and 723 in-hospital deaths after L. Included/excluded descriptors and the distinction between missing chart coverage and inactive treatment intervals are reported in Table S1. Incomplete chart coverage was not synonymous with treatment interruption; the records could not establish whether gaps reflected clinical instability, treatment discontinuation, or documentation practice. Baseline characteristics are summarized in Table 1.

Values are median [Q1, Q3] or *n* (%), from the original pre-imputation cohort. Categorical percentages use N = 1453; continuous summaries use observed values. The primary adjusted analysis used 50 multiply imputed datasets. Missingness was substantial for CRRT mode (34.3%), lactate (33.7%), custom SOFA (37.0%), and 6 h urine output (22.7%); see Table S7. Exact missing counts are reported. SOFA total is missing when any component is unavailable; it was reconstructed after component-wise imputation. The binary suspected-infection field is based on pre-t0 antibiotic–culture pairing, not a validated sepsis diagnosis. Charlson/comorbidity indicators use whole-admission administrative diagnoses. t0, CRRT initiation; BUN, blood urea nitrogen; CRRT, continuous renal replacement therapy; CVVHDF, continuous venovenous hemodiafiltration; MAP, mean arterial pressure; SOFA, Sequential Organ Failure Assessment.

Exposure processing retained 18,032 records after excluding 17 outside active CRRT intervals; no identical-time duplicates or unresolved latest-record ties occurred. Retained records included 51 negative values in 14 patients and 353 warning-flagged values in 166 patients. The cohort had 10, 11, and 12 represented bins in 194, 307, and 952 patients, respectively. In 873 patients, more than one distinct entry occurred in at least one bin, distinguishing the primary record-weighted mean from the alternative bin-based summary (Table S12). Across patients, median charted values were 350 mL [250, 430] for item 224191 (N = 1453), 0 mL [−98.8, 0] for Current Goal (N = 1438), and 337.2 mL [239, 428] for Ultrafiltrate Output (N = 1450). The similar marginal scale of the two removal fields did not imply identical contemporaneous entries or validated delivery (Table S3; Figure S2).

### 3.2. Primary Analysis

Standardized 90-day risks at Q25, the reference, and Q75 were 57.6% (95% CI, 53.7–61.5), 57.5% (54.4–60.5), and 58.6% (54.9–62.3), respectively (Table 2). Compared with 3.79 mL/kg/h, RDs were +0.1 percentage points (−2.4 to +2.6) at 2.73 mL/kg/h and +1.1 percentage points (−2.6 to +4.9) at 5.02 mL/kg/h. Corresponding adjusted ORs were 1.006 (0.887–1.141) and 1.059 (0.878–1.278). Neither the overall association (*p* = 0.475) nor nonlinearity (*p* = 0.505) was statistically detectable. The risk curve and analytic-cohort histogram are shown in Figure 2; the RD curve in Figure S1 shows wider uncertainty toward the exposure tails.

Q25 and Q75 are contrasted with the reference. Overall exposure *p* = 0.475 (3 df); nonlinearity *p* = 0.505 (2 df). Risks and RDs are standardized over the final analytic population. Within-imputation delta-method variances and between-imputation uncertainty were combined with Rubin’s rules on the risk/RD scale, with t-based pointwise intervals; ORs were pooled on the log scale. OR, odds ratio; RD, risk difference; CI, confidence interval; pp, percentage points.

### 3.3. Robustness, Survival, and Secondary Analyses

**Figure 3:**
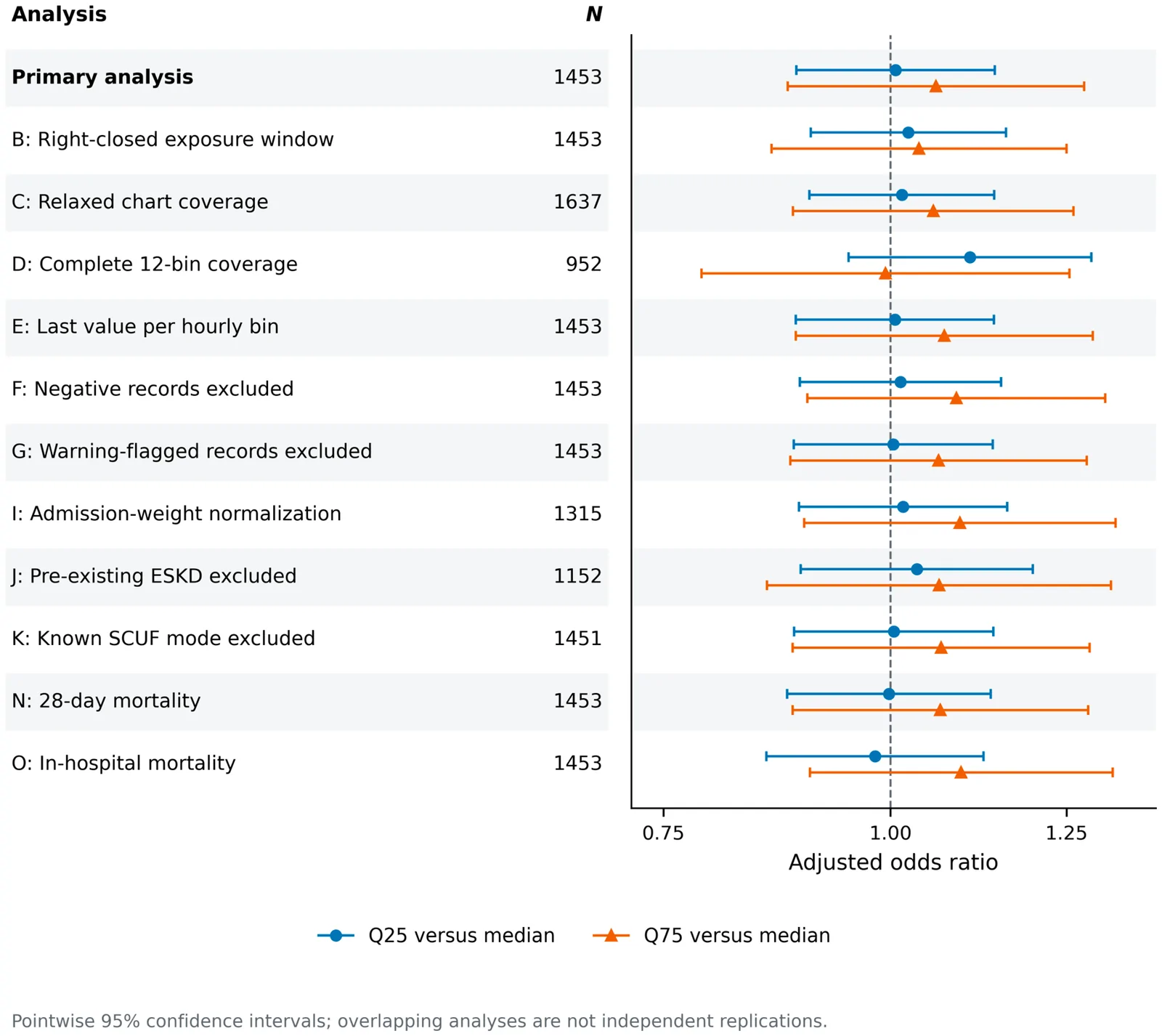
Adjusted ORs at Q25 and Q75 versus the fixed median reference, with pointwise 95% intervals. Each analysis displays its sample size. Full scenario definitions, event counts, and absolute risk contrasts appear in Table S2; Figure S3 provides the corresponding RD forest plot. Specifications overlap and are not independent replications.

For the executed prespecified robustness analyses (B–G and I–K), all reported OR and RD intervals included their null values; overall *p*-values ranged from 0.153 to 0.481 (Figure 3; Table S2). The relaxed-coverage cohort included 1637 patients (*p* = 0.391). The complete 12-bin cohort included 952 patients and had different contrast ordering: Q25 OR 1.106 and RD +2.0 points (95% CI, −1.0 to +5.0), versus Q75 OR 0.994 and RD −0.1 points (−4.8 to +4.5); overall *p* = 0.153. Admission-weight normalization used 1315 patients with 766 deaths (*p* = 0.278); exclusion of pre-existing end-stage kidney disease used 1152 patients with 692 deaths (*p* = 0.295); exclusion of known slow continuous ultrafiltration used 1451 patients with 846 deaths. Exact scenario denominators, event counts, standardized risks, and RDs are reported in Table S2; Figure S3 displays RDs and cohort sizes.

Global Schoenfeld tests rejected proportional hazards in all 50 imputed datasets (all *p* < 0.0001; Table S4). In the Cox sensitivity analysis, overall exposure *p* = 0.067 and nonlinearity *p* = 0.218. Adjusted hazard ratios (HRs) were 1.022 (95% CI, 0.945–1.105) at Q25 and 1.037 (0.924–1.164) at Q75. The pooled exposure-specific time-varying test gave *p* = 0.072. Ninety-day RMST differences were −0.27 days (−2.18 to 1.65) and −0.85 days (−3.91 to 2.22), respectively. The exploratory exposure-by-fluid-balance test gave *p* = 0.627 (Table S4).

For 28-day mortality, the overall association test was *p* = 0.429, with ORs of 0.998 (95% CI, 0.877–1.135) at Q25 and 1.065 (0.883–1.285) at Q75. For in-hospital mortality, the overall *p*-value was 0.300, with corresponding ORs of 0.981 (0.855–1.125) and 1.094 (0.903–1.325).

### 3.4. Missing Data, Diagnostics, and Revision-Stage Analyses

CRRT mode, lactate, the observed custom SOFA total, and 6 h urine output were missing in 34.3%, 33.7%, 37.0%, and 22.7%, respectively. The primary imputation object had no logged events or residual missingness; final mean potential scale-reduction summaries ranged from 0.996 to 1.177. Missingness and observed/imputed distributions are detailed in Tables S7–S9; selected chain traces are shown in Figure S4. The imputed lactate median was 1.8 mmol/L versus an observed median of 2.3, and the reconstructed SOFA median was 12 versus 15. These distributional differences do not validate the imputation assumptions. All 50 primary-model fits converged with finite coefficients and full-rank design matrices; reproduced estimates matched the archived primary results to numerical precision. The median apparent Brier score was 0.198 (constant-risk reference, 0.243). Apparent calibration is not independent validation; influence and design-collinearity summaries are reported in Table S10 and Figure S5.

Complete-case analysis included 361 patients (208 deaths). Ordinary maximum-likelihood fitting converged with finite estimates and a full-rank design; a formal separation-detection test was not performed. Overall *p* = 0.195 and nonlinearity *p* = 0.142. At Q75, OR was 1.307 (95% CI, 0.848–2.015) and RD +4.2 points (−2.5 to +11.0), reflecting greater uncertainty in this selected subcohort. With nonlinear terms for five continuous covariates, overall *p* = 0.479 and nonlinearity *p* = 0.524; Q75 OR was 1.056 (0.872–1.280) and RD +1.1 points (−2.7 to +4.8). Full contrasts and fit summaries are reported in Tables S2 and S11.

## 4. Discussion

In this 12 h landmark cohort, early chart-recorded patient fluid-removal intensity had no statistically detectable adjusted association with 90-day mortality. The primary Q75-versus-median RD was +1.1 percentage points, with a 95% interval from −2.6 to +4.9. This provides a contrast-specific estimate of uncertainty in the central exposure range. These findings do not establish equivalence. Uncertainty was greater in the exposure tails and in the selected complete-case population, whose Q75 interval extended to an 11-point higher risk.

Operationally, item 224191 records an hourly patient-fluid-removal entry during CRRT. Its label and larger positive scale, compared with a Current Goal distribution centered near zero, are compatible with a device-removal quantity rather than the patient’s net-balance target. This interpretation concerns the chart pattern; how clinicians entered the field remains unverified. In prior UFNET research, machine net removal is calculated as effluent minus dialysate and replacement fluids and is distinct from patient balance after all inputs and other outputs [5,9,11,12,13,14,15]. Our record-weighted average lacked validation against delivered volumes, concurrent inputs, or a documented comparator interval. The median of 3.79 mL/kg/h therefore cannot be equated numerically with lower UFNET rates reported using different denominators and windows. Local practice, inputs, documentation, weight choice, and averaging are possible explanations for the difference, but their contributions remain unestablished.

This study contributes a reproducibly specified first 12 h MIMIC-IV chart measure with aligned follow-up, explicit recording-quality evidence, and absolute-risk uncertainty for a defined survivor cohort. The prior literature addresses related questions using heterogeneous designs: Murugan et al. examined fluid-overloaded patients across renal-replacement modalities and a RENAL trial cohort; Tehranian et al. summarized removal across CRRT treatment days; Naorungroj et al. assessed the first 48 h; and Sansom et al. used minute-level machine data from the first 72 h [4,5,6,7,8,9]. Several studies used continuous models, splines, early-death handling, or time-varying analyses. The contribution here is therefore specific to the operational measure and population, with a measurement caution for reuse of this chart field. It neither establishes methodological novelty from those approaches alone nor attributes earlier associations to bias or refutes delivered-UFNET findings.

The landmark aligns completion of the exposure window with the start of follow-up and defines a population meeting recorded 12 h eligibility criteria. Within that window, evolving hemodynamics can affect subsequent removal and prognosis, while prior removal can affect hemodynamics. This treatment–confounder feedback remains unresolved by baseline adjustment. Later fluid strategies, vasopressors, recovery, and organ support were not routinely included as primary covariates because they occur after the exposure window and may lie downstream of early treatment; indiscriminate adjustment could also distort the association. Their causal roles remain uncertain, and the study estimates an adjusted association rather than a causal total treatment effect.

The RELIEVE-AKI feasibility trial illustrates the difficulty of separating removal strategies in practice: hemodynamic instability, rescue removal, and clinician override limited separation between restrictive and liberal delivered UFNET strategies [29]. It studied a different exposure and was not powered to determine mortality effects. RENAL used 90-day mortality, supporting a clinically recognizable fixed horizon for comparison [5,30]; this horizon also permits ascertainment of recorded post-discharge deaths. The 90-day horizon cannot isolate early fluid removal from later illness and treatment. The 28-day and in-hospital analyses provide complementary shorter-horizon results while remaining subject to later influences. Trials of renal-replacement solute-clearance intensity [30,31] address a separate treatment dimension and do not establish a UFNET target.

The survival analyses complement the fixed-horizon mortality analysis. Cox regression uses event timing, whereas logistic regression addresses mortality by a specified horizon; their overall *p*-values therefore need not agree. The exposure-specific proportionality test concerns only the exposure terms. With global proportional-hazards violations, the Cox HRs remain secondary model-dependent summaries. RMST provides a complementary survival-time contrast without requiring proportional hazards, although the direct regression still depends on recorded mortality ascertainment and model specification.

Strengths include a large critical-care cohort, fixed exposure anchors, alignment of follow-up with completion of the exposure window, and continuous exposure modeling without arbitrary categories. An internally dated analysis plan, explicit cohort and endpoint algorithms, 50-imputation handling of missing covariates with reported diagnostics, and absolute-risk contrasts alongside ORs make the analysis reproducible and its uncertainty interpretable. Data-free code and computational checks support reproduction of the primary estimates, as detailed in the Supplementary Materials. All executed prespecified analyses are reported, including differences in contrast ordering. The plan-declared complete-case sensitivity was reassessed and reported in detail during revision; the covariate-spline sensitivity was specified at revision.

### Limitations

This single-center observational study remains susceptible to residual confounding, confounding by indication, and within-window treatment adaptation. Administrative diagnosis codes were not time-stamped before CRRT and may imperfectly represent baseline comorbidity; the adjustment set therefore cannot be described as wholly measured before t0. Eligibility depends on recorded administrative and clinical times, which may be discordant and permit selection misclassification. Landmark conditioning and chart-coverage requirements additionally select survivors with sufficient documentation (Table S1), limiting applicability to early deaths or discontinuations. The charted exposure was not independently validated as delivered UFNET and does not reconstruct total patient fluid balance or treatment downtime. Variation in documentation frequency, together with record weighting that allows hours with more entries to contribute more heavily, may add measurement variability to the primary exposure.

High covariate missingness makes inference dependent on the imputation assumptions; satisfactory computational diagnostics cannot demonstrate missing at random. Complete cases represent a smaller selected population with different covariate standardization and wider uncertainty. Mortality classification depends on death-record linkage; unrecorded deaths would be misclassified, and no separate loss-to-follow-up process was modeled. Intervals are pointwise, and overlapping nominal tests are not independent confirmations; global proportional-hazards violations limit interpretation of the secondary Cox HRs. Finally, the original plan has internal provenance rather than external registration, and the planned post-t0 sensitivity was not operationally executable.

## 5. Conclusions

Among adults meeting recorded 12 h landmark eligibility during CRRT, early weight-normalized intensity derived from chart-recorded values in the “Hourly Patient Fluid Removal” field was not detectably associated with subsequent 90-day mortality. Absolute-risk intervals quantify uncertainty at specified exposure contrasts and remain compatible with clinically relevant differences. The findings concern this chart-defined measure and selected cohort; they do not establish equivalence of treatment intensities, the causal effect of delivered UFNET, or an optimal removal target.

## Figures and Tables

**Table 1:** Baseline characteristics of the final landmark cohort.

Characteristic	Overall (N = 1453)	Missing, *n* (%)
Chart-recorded hourly patient fluid-removal intensity, mL/kg/h	3.79 [2.73, 5.02]	0 (0.0%)
Age at t0, years	64 [53, 74]	0 (0.0%)
Sex–Male	896 (61.7%)	0 (0.0%)
Female	557 (38.3%)	—
Race group–White	865 (59.5%)	0 (0.0%)
Asian	36 (2.5%)	—
Black	160 (11.0%)	—
Hispanic/Latino	61 (4.2%)	—
Other/unknown	331 (22.8%)	—
Admission type–EW emergency	610 (42.0%)	0 (0.0%)
Urgent	444 (30.6%)	—
Observation admission	214 (14.7%)	—
Direct emergency	78 (5.4%)	—
Same-day surgical admission	66 (4.5%)	—
Elective	41 (2.8%)	—
First ICU type–Medical Intensive Care Unit (MICU)	542 (37.3%)	0 (0.0%)
Cardiac Vascular Intensive Care Unit (CVICU)	266 (18.3%)	—
Coronary Care Unit (CCU)	173 (11.9%)	—
Medical/Surgical Intensive Care Unit (MICU/SICU)	165 (11.4%)	—
Surgical Intensive Care Unit (SICU)	183 (12.6%)	—
Trauma SICU (TSICU)	102 (7.0%)	—
Other	22 (1.5%)	—
Calendar period–2008–2010	400 (27.5%)	0 (0.0%)
2011–2013	287 (19.8%)	—
2014–2016	285 (19.6%)	—
2017–2019	365 (25.1%)	—
2020–2022	116 (8.0%)	—
ICU admission to CRRT initiation, hours	47 [19, 107]	0 (0.0%)
CRRT mode–CVVHDF	901 (62.0%)	499 (34.3%)
Other known	53 (3.6%)	—
Charlson comorbidity index	4 [2, 5]	0 (0.0%)
Chronic kidney disease–Yes	698 (48.0%)	0 (0.0%)
No	755 (52.0%)	—
Heart failure–Yes	653 (44.9%)	0 (0.0%)
No	800 (55.1%)	—
Chronic liver disease–Yes	460 (31.7%)	0 (0.0%)
No	993 (68.3%)	—
Malignancy–Yes	190 (13.1%)	0 (0.0%)
No	1263 (86.9%)	—
Custom pre-CRRT SOFA total	15 [14, 17]	537 (37.0%)
Documented suspected infection before t0–Yes	1225 (84.3%)	0 (0.0%)
No	228 (15.7%)	—
Mechanical ventilation at t0–Yes	1126 (77.5%)	0 (0.0%)
No	327 (22.5%)	—
Norepinephrine-equivalent dose at t0, µg/kg/min	0.10 [0.00, 0.28]	0 (0.0%)
Median MAP during [t0−6 h, t0), mmHg	71 [66, 78]	2 (0.1%)
Closest lactate during [t0−6 h, t0), mmol/L	2.3 [1.4, 5.2]	489 (33.7%)
Closest pH during [t0−6 h, t0)	7.32 [7.25, 7.38]	64 (4.4%)
Closest creatinine during [t0−24 h, t0), mg/dL	4.0 [2.9, 5.4]	13 (0.9%)
Closest BUN during [t0−24 h, t0), mg/dL	63 [41, 91]	13 (0.9%)
Closest bicarbonate during [t0−24 h, t0), mEq/L	19 [15, 22]	15 (1.0%)
Closest potassium during [t0−24 h, t0), mEq/L	4.7 [4.1, 5.4]	14 (1.0%)
Urine output during [t0−6 h, t0), mL/kg	0.68 [0.19, 2.20]	330 (22.7%)
Cumulative ICU-admission-to-t0 fluid balance, mL/kg	84.4 [28.3, 165.0]	105 (7.2%)
Number of represented exposure bins	12 [11, 12]	0 (0.0%)
Represented exposure bins–10	194 (13.4%)	0 (0.0%)
11	307 (21.1%)	—
12	952 (65.5%)	—

**Table 2:** Standardized 90-day mortality risks and adjusted contrasts (N = 1453; 847 deaths; 50 imputations).

Contrast	Exposure, mL/kg/h	Standardized Risk (95% CI)	Risk Difference (95% CI)	Adjusted OR (95% CI)
Q25	2.73 vs. 3.79	57.6% (53.7–61.5)	+0.1 pp (−2.4 to +2.6)	1.006 (0.887–1.141)
Reference	3.79	57.5% (54.4–60.5)	Reference	1.000
Q75	5.02 vs. 3.79	58.6% (54.9–62.3)	+1.1 pp (−2.6 to +4.9)	1.059 (0.878–1.278)

## Data Availability

MIMIC-IV v3.1 is available through PhysioNet (https://doi.org/10.13026/kpb9-mt58), subject to credentialing, required training, and the Data Use Agreement. Restricted patient-level data cannot be redistributed. Only Hasan Burak Toprak, the credentialed investigator and corresponding author, accessed and analyzed those data. Gürhan Taşkın and Hüseyin Levent Yamanel reviewed the protocol, code logic, non-patient-level aggregate outputs, interpretation, and manuscript without accessing restricted patient-level data. Data-free analysis code, variable definitions, the analysis plan, and aggregate supporting tables accompany this revision.

## Supplementary Materials

The following supporting information can be downloaded at: https://www.mdpi.com/article/10.3390/medicina62101913/s1, Table S1: Selection and recording coverage; Table S2: Logistic analyses with standardized risks and contrasts; Table S3: Descriptive chart-field comparison; Table S4: Survival and exploratory results; Table S5: Covariate definitions and model dimensions; Table S6: Imputation quality control for original scenario runs; Table S7: Missingness by outcome and exposure quartile; Table S8: Primary imputation methods and predictor roles; Table S9: Observed and imputed distributions and convergence; Table S10: Primary model diagnostics; Table S11: Complete-case and covariate-spline sensitivity details; Table S12: Exposure processing and distribution; Figure S1: Exposure distribution and standardized risk difference; Figure S2: Patient summaries of charted fluid-removal fields; Figure S3: Absolute risk differences across analyses; Figure S4: Multiple imputation chain traces; Figure S5: Apparent calibration; Supplementary_Data_Medicina.zip: aggregate tables, exposure-range counts, figure data, and imputation predictor matrix; Code_and_Aggregate_Support_Medicina.zip: data-free code, variable dictionary, original analysis plan, pre-outcome amendment log, and retrospective implementation history.
