# Supplementary material for: Chart-Recorded Hourly Patient Fluid-Removal Intensity and 90-Day Mortality After a 12-Hour Landmark During Continuous Renal Replacement Therapy
Source: Medicina (Kaunas). 2026 Oct 3;62(10):1913. doi: 10.3390/medicina62101913 (PMC13706772; doi:10.3390/medicina62101913)
Supplement: Supplementary file 1 [file medicina-62-01913-s001.zip › medicina-4573882-supplementary.pdf]

Supplementary Materials

Chart-Recorded Hourly Patient Fluid-Removal Intensity and 90-Day Mortality After a 12-Hour Landmark During Continuous Renal Replacement Therapy

Hasan Burak Toprak, Gürhan Taşkın and Hüseyin Levent Yamanel

This supplement provides the operational definitions, absolute-risk results, recording-quality summaries, selection characteristics, imputation information and model diagnostics supporting the manuscript. The original primary analysis is retained. Complete-case sensitivity was declared in the original analysis plan and was reassessed and reported in detail during revision; the covariate-spline sensitivity was specified during revision.

Supplementary Methods

Exposure construction

The exposure used MIMIC-IV chartevents item 224191, "Hourly Patient Fluid Removal" (abbreviation "Hourly PFR"), a numeric field in the Dialysis category, recorded in mL during a documented active CRRT interval in [t0, t0 + 12 h). Hourly bins were anchored at t0. Same-charttime values were ordered by latest storetime; an unresolved latest-storetime tie with discordant values was excluded. The primary summary was the arithmetic mean of retained signed values divided by analytic body weight. This is a record-weighted mean, not the sum of hourly volumes divided by elapsed treatment time. The primary coverage rule required at least 10 represented bins and at least four in each 6-hour half. Exposure was not imputed. The dictionary label specifies an hourly quantity, but agreement with delivered device UFNET was not independently established.

The index CRRT initiation was the earliest procedureevents row per patient with item 225802, 225803, 225809 or 225955, nonmissing start and end times, and end time later than start time. Ties were ordered by start time, ICU stay, end time and item identifier. Overlapping or touching valid CRRT intervals within that index stay were combined; gaps were retained. Later rows in the index stay contributed activity intervals but did not create additional observations, and later ICU stays were not substituted as index stays. All final-cohort index rows used item 225802 (Dialysis–CRRT).

The prespecified knot rule used the 5th, 35th, 65th and 95th percentiles of the pre-outcome exposure distribution (N = 1465), giving 1.2912, 3.1650, 4.4765 and 7.0493 mL/kg/h. The reference was 3.7932, with fixed contrasts at 2.7282 and 5.0167 mL/kg/h. These original constants were retained for all reported contrasts. The final analytic-cohort median is 3.7917; its slight difference from the fixed reference does not change the contrast definition. The fitted-curve display limits were the fixed pre-outcome 1st and 99th percentiles, 0.4197 and 9.1308 mL/kg/h (N = 1465). These are distinct from the final-cohort percentiles reported descriptively in Table S12 (N = 1453); observations outside the display limits remained in the models.

Landmark and outcome timing

The landmark was L = t0 + 12 h. Eligibility required no precise hospital death timestamp at or before L. If a hospital timestamp was unavailable, a death date before the landmark date excluded the patient, and a death date equal to the landmark date was temporally indeterminate. Continued hospital observation was

operationalized using the recorded hospital discharge timestamp, with a timestamp at or before L classified as ineligible. Follow-up began at L. Figure 1 reports the combined 12 landmark exclusions.

The fixed 90-day and 28-day outcomes used patients.dod only: a nonmissing death date on or before DATE(L) + 90 or DATE(L) + 28, respectively. The hospital death timestamp did not override patients.dod for these binary endpoints. No recorded death within the horizon was coded as no recorded death, conditional on the completeness of the database linkage. In-hospital mortality required admissions.deathtime > L. The hospital-expire flag was used only as a quality-control check.

Imputation and inference

The primary imputation used 50 imputations, 50 iterations and seed 42. Continuous incomplete covariates used predictive mean matching; SOFA components used proportional-odds imputation except the cardiovascular component, which used multinomial regression under the documented imputation-specification amendment. CRRT mode used binary logistic imputation. Total SOFA was reconstructed deterministically from the six completed component labels after imputation; it was not itself imputed within mice. The outcome, event-time and three exposure-basis variables were predictors and were not imputed. The complete predictor matrix is supplied in Supplementary_Data/MI_PREDICTOR_MATRIX_FROM_OBJECT.tsv.

Separate original imputation runs were used for B, C, E, F, G, I and O. Each exposure variant replaced the primary exposure spline predictors; the in-hospital-outcome run added that fully observed outcome as an imputation predictor. D, J and K applied cohort restrictions to the primary completed datasets. N reused primary covariate imputations for the 28-day outcome. The revision used these stored imputations without running MICE again.

Within each completed dataset, standardized risks were obtained by setting the exposure to each contrast value and averaging predicted probabilities over the analysis population. Delta-method variances used the full fitted coefficient covariance; risk differences used the joint contrast gradient. Risk and risk-difference estimates were pooled directly on their respective scales using Rubin's rules with t-based confidence intervals. OR contrasts were pooled on the log-OR scale. The complete-case analysis used its own population for standardization, Wald/delta-method confidence intervals and likelihood-ratio tests; its risks therefore target a selected complete-case covariate distribution.

All inferential tests are nominal, without multiplicity correction. The analyses overlap and do not constitute independent replications. The original internal analysis plan, its pre-outcome amendment/deviation log and a retrospective implementation history are supplied with the code materials. These internal records are not an externally registered protocol or independently time-stamped preregistration.

Earlier internal work using item 225183 ("Current Goal") was superseded by the present item-224191 study, and none of its results are used in this manuscript. The pre-outcome designation in the supplied analysis plan refers to the restarted item-224191 study.

After outcome access but before the first submission, implementation amendments aligned fixed-horizon mortality with patients.dod as specified in SAP Section 8, repaired respiratory SOFA PaO2–FiO2 pairing, changed cardiovascular SOFA imputation from proportional-odds (polr) to multinomial regression (polyreg), and corrected Cox contrast extraction to select the exposure coefficients by name. The first

submitted manuscript already used the corrected results. The retrospective history distinguishes these changes from the original plan and revision-stage work (docs/IMPLEMENTATION_HISTORY.md).

Computational verification. Checks comprised matching source-file identities, reviewing extraction and modeling code, reconciling intermediate selection counts, independently recomputing aggregate cohort and exposure summaries from the source tables, confirming row alignment, and reproducing the primary estimates using the existing imputations. These were computational reproducibility and consistency checks within the study workflow, not independent clinical validation of chart entries or replication by an external analyst.

Prespecified analysis definitions

ID	Definition	Role/status
A	Adjusted 90-day Cox model, formal exposure-specific PH test, and 90-day RMST	Sensitivity
B	Alternative exposure boundary (t0,t0+12 h]	Sensitivity
C	Relaxed exposure coverage: ≥8 bins and ≥3 bins per 6-hour half	Sensitivity
D	Complete 12-bin cohort	Sensitivity
E	Last valid record per bin, then mean across bins	Sensitivity
F	Exclude negative exposure records	Sensitivity
G	Exclude warning-flagged exposure records	Sensitivity
H	Procedure-weight-only definition	Redundant by construction; not modeled
I	Admission-weight-only exposure; exclude missing exposure	Sensitivity
J	Exclude pre-existing ESKD/maintenance dialysis	Sensitivity
K	Exclude known SCUF index mode	Sensitivity; unknown CRRT mode retained
L	Additional post-t0 MAP/vasopressor adjustment	Prespecified but not executable; operational detail insufficient
M	Exposure × pre-t0 fluid-balance interaction	Exploratory
N	28-day mortality	Secondary outcome
O	In-hospital mortality	Secondary outcome
CC	Complete-case sensitivity with the primary exposure spline and adjustment set	Declared in SAP Section 10; reassessed and reported in detail during revision

Complete-case sensitivity was declared in SAP Section 10. Earlier provisional complete-case calculations were reassessed during revision, with expanded diagnostics and detailed reporting. The joint covariate-spline sensitivity was specified during

revision. Neither replaces the original primary model. The proposed post-t0 adjustment (L) was not executed because the required time-varying variables had not been specified in sufficient operational detail or built for that analysis.

Table S1 Selection and recording coverage

Panel A Sequential participant flow

Stage	Number remaining or excluded
Patients with at least one valid CRRT procedure row	2095
Excluded for not active at L or no valid analytic weight	237
Remaining after activity and weight criteria	1858
Excluded at age ICU-stay timing or coverage step	393
Pre-outcome cohort meeting primary criteria	1465
Combined landmark exclusions for death discharge or indeterminate timing	12
Final analytic cohort	1453

Panel B Available group characteristics

Characteristic	Not active at L	Age timing or coverage step	Combined landmark exclusions	Included
N	236	393	12	1453
Age years median [IQR]	65 [55-75]	63 [53-74]	56 [50-74]	64 [53-74]
Female n (%)	98 (41.5%)	147 (37.4%)	4 (33.3%)	557 (38.3%)
White race n (%)	141 (59.7%)	228 (58.0%)	4 (33.3%)	865 (59.5%)
Emergency admission n (%)	131 (55.5%)	164 (41.7%)	6 (50.0%)	688 (47.4%)
First ICU MICU n (%)	84 (35.6%)	68 (17.3%)	6 (50.0%)	542 (37.3%)
Period 2008–2013 n (%)	128 (54.2%)	174 (44.3%)	3 (25.0%)	687 (47.3%)
ICU admission to t0 h median [IQR]	24 [10-63]	33 [13-98]	54 [14-72]	47 [19-107]
Analytic weight available n (%)	236 (100.0%)	393 (100.0%)	12 (100.0%)	1453 (100.0%)
Represented bins median [IQR]	5 [3-8]	8 [6-9]	12 [12-12]	12 [11-12]
Recorded death by t0 + 90 d n (%)	173 (73.3%)	237 (60.3%)	12 (100.0%)	847 (58.3%)
Index-admission hospital death n (%)	158 (66.9%)	207 (52.7%)	11 (91.7%)	723 (49.8%)

These are descriptive comparisons of available common variables, not hypothesis tests. The one patient excluded for unavailable analytic weight is included in the sequential flow but is not individually characterized. The descriptive mortality column is measured from CRRT initiation (t0), not from L, and is not the primary estimand. Hospital death includes the index admission and is not restricted to post-landmark deaths for excluded groups. The combined landmark row retains all 12 exclusions without separate component groups. SOFA and laboratory comparisons are not available uniformly across the earlier exclusion stages.

Panel C Coverage failures

Descriptor among 387 coverage failures	Value
Represented bins median [IQR]	8 [6-9]
No retained exposure record	4
Active bins without a chart record median [IQR]	4 [3-5]
At least one active bin without a record	379
Bins without active CRRT median [IQR]	0 [0-0]
At least one bin without active CRRT	61
Fully active CRRT but gaps in charting	326

Coverage-failure descriptors overlap and are not additive. Recording gaps can coexist with treatment interruption. Coverage failures are a subset of the combined age/timing/coverage step and should not be equated with all exclusions. Among the 387 coverage failures, 326 had recorded active CRRT throughout all 12 bins but gaps in item-224191 charting, and 61 had at least one bin without recorded active CRRT. These records cannot establish whether the underlying reason was clinical instability, treatment discontinuation or documentation practice.

Table S2 Logistic analyses with standardized risks and contrasts

Panel A Analysis populations and tests

Analysis	N	Deaths	Overall P	Nonlinear P
Primary	1453	847	0.4749	0.5050
B Boundary	1453	847	0.4683	0.5969
C Relaxed coverage	1637	953	0.3913	0.4478
D Complete 12 bins	952	569	0.1530	0.4174
E Last per bin	1453	847	0.4249	0.4354
F Negative records removed	1453	847	0.2804	0.2851
G Warning records removed	1453	847	0.4809	0.4957
I Admission weight	1315	766	0.2775	0.2754
J ESKD excluded	1152	692	0.2946	0.3836

Analysis	N	Deaths	Overall P	Nonlinear P
K Known SCUF excluded	1451	846	0.4555	0.4664
N 28-day death	1453	719	0.4290	0.4557
O In-hospital death	1453	723	0.2995	0.2990
CC Complete case	361	208	0.1954	0.1424
Covariate splines	1453	847	0.4787	0.5244

The primary and all logistic sensitivities use 90-day mortality except N and O. Q25 = 2.7282, reference = 3.7932 and Q75 = 5.0167 mL/kg/h. Overall/nonlinearity tests use 3/2 df; pooled D1 tests are used for MI models and likelihood-ratio tests for CC. CC denotes the plan-declared complete-case sensitivity reassessed and reported in detail during revision; the covariate-spline analysis was specified during revision.

Panel B Standardized mortality risks

Analysis	Risk at Q25 % (95% CI)	Risk at reference % (95% CI)	Risk at Q75 % (95% CI)
Primary	57.6 (53.7 to 61.5)	57.5 (54.4 to 60.5)	58.6 (54.9 to 62.3)
B Boundary	58.0 (54.0 to 62.0)	57.6 (54.5 to 60.7)	58.3 (54.7 to 61.8)
C Relaxed coverage	57.5 (53.9 to 61.2)	57.2 (54.4 to 60.1)	58.3 (54.9 to 61.8)
D Complete 12 bins	60.5 (55.7 to 65.2)	58.5 (54.7 to 62.3)	58.4 (53.9 to 62.8)
E Last per bin	57.5 (53.5 to 61.4)	57.4 (54.2 to 60.5)	58.7 (55.1 to 62.3)
F Negative records removed	57.3 (53.4 to 61.2)	57.0 (54.0 to 60.1)	58.7 (55.0 to 62.4)
G Warning records removed	57.6 (53.7 to 61.4)	57.5 (54.4 to 60.5)	58.7 (55.0 to 62.4)
I Admission weight	57.1 (53.0 to 61.3)	56.8 (53.6 to 60.1)	58.6 (54.8 to 62.4)
J ESKD excluded	59.5 (55.2 to 63.7)	58.8 (55.5 to 62.2)	60.0 (55.8 to 64.2)
K Known SCUF excluded	57.5 (53.6 to 61.4)	57.4 (54.4 to 60.5)	58.7 (55.0 to 62.4)
N 28-day death	48.7 (44.8 to 52.6)	48.7 (45.6 to 51.8)	50.0 (46.1 to 53.9)
O In-hospital death	48.6 (44.6 to 52.6)	49.0 (45.9 to 52.1)	50.8 (46.8 to 54.9)
CC Complete case	54.4 (47.4 to 61.5)	58.6 (52.9 to 64.3)	62.8 (55.7 to 69.9)
Covariate splines	57.7 (53.8 to 61.5)	57.5 (54.4 to 60.5)	58.6 (54.9 to 62.2)

Panel C Absolute risk differences

Analysis	Q25 minus reference percentage points (95% CI)	Q75 minus reference percentage points (95% CI)
Primary	+0.1 (-2.4 to +2.6)	+1.1 (-2.6 to +4.9)

Analysis	Q25 minus reference percentage points (95% CI)	Q75 minus reference percentage points (95% CI)
B Boundary	+0.4 (-2.0 to +2.9)	+0.7 (-3.0 to +4.4)
C Relaxed coverage	+0.3 (-2.0 to +2.6)	+1.1 (-2.4 to +4.5)
D Complete 12 bins	+2.0 (-1.0 to +5.0)	-0.1 (-4.8 to +4.5)
E Last per bin	+0.1 (-2.4 to +2.6)	+1.4 (-2.4 to +5.1)
F Negative records removed	+0.3 (-2.3 to +2.8)	+1.7 (-2.1 to +5.4)
G Warning records removed	+0.1 (-2.4 to +2.6)	+1.2 (-2.5 to +4.9)
I Admission weight	+0.3 (-2.3 to +2.9)	+1.8 (-2.2 to +5.7)
J ESKD excluded	+0.6 (-2.2 to +3.5)	+1.2 (-3.0 to +5.3)
K Known SCUF excluded	+0.1 (-2.4 to +2.6)	+1.3 (-2.5 to +5.0)
N 28-day death	+0.0 (-2.7 to +2.6)	+1.3 (-2.5 to +5.1)
O In-hospital death	-0.4 (-3.2 to +2.4)	+1.8 (-2.1 to +5.7)
CC Complete case	-4.1 (-9.2 to +1.0)	+4.2 (-2.5 to +11.0)
Covariate splines	+0.2 (-2.3 to +2.7)	+1.1 (-2.7 to +4.8)

Panel D Adjusted odds ratios

Analysis	Q25 vs reference OR (95% CI)	Q75 vs reference OR (95% CI)
Primary	1.0064 (0.8874 to 1.1413)	1.0593 (0.8778 to 1.2783)
B Boundary	1.0229 (0.9040 to 1.1575)	1.0368 (0.8600 to 1.2499)
C Relaxed coverage	1.0145 (0.9024 to 1.1406)	1.0557 (0.8838 to 1.2611)
D Complete 12 bins	1.1060 (0.9484 to 1.2899)	0.9936 (0.7870 to 1.2546)
E Last per bin	1.0056 (0.8868 to 1.1402)	1.0706 (0.8869 to 1.2925)
F Negative records removed	1.0129 (0.8916 to 1.1507)	1.0870 (0.9001 to 1.3127)
G Warning records removed	1.0035 (0.8847 to 1.1382)	1.0629 (0.8808 to 1.2826)
I Admission weight	1.0161 (0.8906 to 1.1592)	1.0920 (0.8963 to 1.3304)
J ESKD excluded	1.0339 (0.8924 to 1.1978)	1.0634 (0.8551 to 1.3225)
K Known SCUF excluded	1.0041 (0.8851 to 1.1392)	1.0662 (0.8832 to 1.2871)
N 28-day death	0.9980 (0.8772 to 1.1354)	1.0653 (0.8834 to 1.2848)
O In-hospital death	0.9806 (0.8546 to 1.1252)	1.0937 (0.9028 to 1.3251)

Analysis	Q25 vs reference OR (95% CI)	Q75 vs reference OR (95% CI)
CC Complete case	0.7741 (0.5623 to 1.0656)	1.3070 (0.8477 to 2.0151)
Covariate splines	1.0093 (0.8881 to 1.1471)	1.0564 (0.8717 to 1.2803)

Risks and risk differences are standardized within each analysis population; cross-scenario differences may therefore reflect population changes as well as exposure definitions. D has a different Q25/Q75 point-estimate ordering, and CC has wider uncertainty. All contrast intervals include the null. Exact scenario counts replace the former author-applied blanket small-cell suppression. The machine-readable table retains the available source precision; four-decimal source rounding in historical sensitivities should not be interpreted as measurement precision.

Table S3 Descriptive chart-field comparison

Metric	225183 Current Goal	226457 Ultrafiltrate Output
unit of 224191 / comparator	mL / mL	mL / mL
represented interval documented for comparator	Not documented	Not documented
valid comparator records in window	17785	17325
patients with >=1 valid comparator record	1438	1450
synchronous 224191-comparator pairs (exact charttime, same stay)	15861	13672
bins with >1 synchronous pair (latest pair kept)	980	122
matched hour bins after latest-per-bin rule	14826	13550
patients with >=1 matched bin (overlap population)	1438	1445
patients with 0 matched bins	15	8
matched bins per patient in overlap population: median [IQR]; p10-p90	11 [10-12]; 7-12	10 [8-12]; 4-12
share of patient's represented 224191 bins matched: median [IQR]	1.000 [0.900-1.000]	0.917 [0.727-1.000]
224191 patient medians: median [IQR]; p05-p95	350.0 [250.0-430.0]; 100.0-600.0	350.0 [250.0-430.0]; 100.0-600.0
Comparator patient medians: median [IQR]; p05-p95	0.0 [-98.8-0.0]; -200.0-150.0 (n=1438)	337.2 [239.0-428.0]; 100.0-573.0 (n=1450)
share of matched bins with identical recorded numbers, per patient: median [IQR]	0.000 [0.000-0.000]	0.000 [0.000-0.083]
patients with all matched bins identical / none identical	5 / 1203 (of 1438)	3 / 980 (of 1445)

All three fields belong to chartevents and have recorded mL units. Comparisons used the same ICU stay and active-CRRT window, exact charttime matches, and the latest synchronous pair per anchored hourly bin. Patient medians summarize repeated records before summarizing across patients. Matching

timestamps does not prove that fields refer to the same measurement interval. Comparator intervals were not documented; no conversion to hourly rates, ICC, Bland–Altman limits or numeric difference analysis was performed. Identical recorded numbers are documentation descriptors, not agreement validation. The pattern is compatible with different roles for patient fluid-removal recording and fluid-balance goals, but it cannot determine achieved device removal, concurrent-intake compensation or construct equivalence to delivered UFNET.

Table S4 Survival and exploratory results

Analysis	Contrast or test	Estimate or P value
Cox overall exposure test	3 df	P=0.0666
Cox nonlinearity test	2 df	P=0.2184
Cox HR, Q25 vs reference	2.7282 vs 3.7932	1.0220 (0.9451–1.1052)
Cox HR, Q75 vs reference	5.0167 vs 3.7932	1.0373 (0.9244–1.1640)
Formal exposure-specific PH test	3 df	P=0.0724
RMST difference, Q25 minus reference	τ=90 days	−0.27 days (−2.18 to 1.65)
RMST difference, Q75 minus reference	τ=90 days	−0.85 days (−3.91 to 2.22)
Exposure × pre-t0 fluid-balance interaction	3 df	P=0.6268

Survival time originated at L = t0 + 12 h. The Cox population was the primary landmark cohort (N = 1453; 847 events; 606 administrative censorings at day 90), with Efron handling of ties and the same exposure spline and 26 adjustment terms. Fractional days were used when a post-landmark hospital death timestamp agreed with the recorded death date; otherwise time was represented in calendar days. All 80 same-calendar-day deaths had usable subsequent timestamps and positive intervals; no arbitrary epsilon was added. No separate loss-to-follow-up censoring was modeled.

Global proportional hazards were assessed with survival::cox.zph using Kaplan–Meier-transformed time (transform="km") and term-level testing (terms=TRUE). The diagnostics were evaluated descriptively within each imputed dataset, were not pooled, and were not used for exposure inference or model selection. The retained summary contains 50 successful diagnostics; the median, minimum, and maximum global P values all round to 0.0000 at four decimal places. This supports all P < 0.0001, rather than exact zero, and the global tests rejected proportional hazards in all 50 datasets. The source summary is supplied as A_COX_ZPH_DESCRIPTIVE_PER_IMPUTATION.tsv. Separately, the formal pooled exposure-specific PH test jointly assessed three spline-by-log(time/28) interactions, yielding P = 0.072; it does not establish proportionality for the full model. Cox HRs are therefore interpreted cautiously as secondary model-dependent summaries. RMST provides a complementary survival summary that does not require proportional hazards.

The 90-day restricted-time outcome equalled the recorded death time for an event and 90 days otherwise. It was regressed directly on the same exposure spline and covariates within each completed dataset using linear regression with HC3 covariance. The fixed exposure contrasts and variances were pooled with

Rubin's rules. This calculation does not require proportional hazards, but depends on the death-ascertainment and regression assumptions; it was not obtained by integrating a Cox survival curve.

Table S5 Covariate definitions and model dimensions

The primary design matrix contained 45 columns including the intercept: 3 exposure columns and 41 adjustment columns from 26 adjustment terms. Continuous adjustment covariates entered linearly in the primary model. Categorical terms used indicator coding. Selection reflected clinical roles in severity, treatment allocation and prognosis, not univariable significance screening. Administrative comorbidity codes cover the index admission and are not timestamped to establish pre-t0 availability. The implemented calendar-period covariate used patients.anchor_year_group, as shown below; the original plan described calendar year.

Variable	Source and measurement window	Unit	Primary model form
Exposure spline	chartevents 224191 (Hourly Patient Fluid Removal, mL); analytic weight; [t0, t0+12h), index stay, active CRRT intervals	mL/kg per charted hour	4-knot RCS (1.2912, 3.1650, 4.4765, 7.0493); 3 columns; 3 column(s)
Age	patients.anchor_age, anchor_year; at t0	years	linear; 1 column(s)
Sex	patients.gender; fixed	M/F	factor M (ref), F; 1 column(s)
Race group	admissions.race; index admission	category	factor, ref WHITE; 4 columns; 4 column(s)
Admission type	admissions.admission_type; index admission	category	factor, ref EW EMER.; 5 columns; 5 column(s)
First ICU type	icustays.first_careunit; index ICU stay	category	factor, ref MICU; 6 columns; 6 column(s)
Calendar period	patients.anchor_year_group; de-identified era	category	factor, ref 2008-2010; 4 columns; 4 column(s)
ICU admission to CRRT start	icustays.intime; t0; intime to t0	hours	linear; 1 column(s)
CRRT mode	chartevents 227290 (CRRT mode, text); fallback index procedure itemid; latest value with charttime <= t0	category	factor CVVHDF (ref) vs OTHER_KNOWN; unknown = missing; 1 column(s)
Charlson index	diagnoses_icd of the index admission (Quan 2005 ICD-9/10); whole index admission (administrative proxy; codes not time-stamped)	points	linear; 1 column(s)
Chronic kidney disease	diagnoses_icd (Charlson renal component); whole index admission (administrative proxy)	0/1	factor 0/1; 1 column(s)

Variable	Source and measurement window	Unit	Primary model form
Heart failure	diagnoses_icd (Charlson CHF component); whole index admission (administrative proxy)	0/1	factor 0/1; 1 column(s)
Chronic liver disease	diagnoses_icd (Charlson mild or severe liver); whole index admission (administrative proxy)	0/1	factor 0/1; 1 column(s)
Malignancy	diagnoses_icd (Charlson malignancy or metastatic); whole index admission (administrative proxy)	0/1	factor 0/1; 1 column(s)
Custom SOFA total	Pre-t0 laboratory/charted measurements; ventilation and norepinephrine-equivalent dose at t0. Component-specific windows: see Table S5 note.	points (0-24)	linear; components imputed, total reconstructed; 1 column(s)
Documented suspected infection before t0	Prescriptions plus microbiology; antibiotic–culture pairing, without a required acute SOFA change; both strictly before t0	0/1	factor 0/1; 1 column(s)
Invasive ventilation at t0	procedureevents 225792; interval covering t0	0/1	factor 0/1; 1 column(s)
Norepinephrine-equivalent dose	inputevents 221906, 221289, 221749, 221662, 222315; infusions active at t0	μg/kg/min	linear; 1 column(s)
MAP	chartevents 220052/220181/225312 (10-250 mmHg); [t0-6h, t0)	mmHg	linear; 1 column(s)
Lactate	labevents 50813 or chartevents 225668; [t0-6h, t0), closest to t0	mmol/L	linear; 1 column(s)
pH	labevents 50820 or chartevents 223830/220274; [t0-6h, t0), closest to t0	pH units	linear; 1 column(s)
Creatinine	labevents 50912; [t0-24h, t0), closest	mg/dL	linear; 1 column(s)
BUN	labevents 51006; [t0-24h, t0), closest	mg/dL	linear; 1 column(s)
Bicarbonate	labevents 50882; [t0-24h, t0), closest	mEq/L	linear; 1 column(s)
Potassium	labevents 50971; [t0-24h, t0), closest	mEq/L	linear; 1 column(s)
Urine output	outputevents urine items; irrigant subtracted (full IDs in data dictionary); [t0-6h, t0) and >= ICU admission	mL/kg per 6 h (total, not per hour)	linear; 1 column(s)
Cumulative fluid balance	inputevents (mL/L amounts, no additives, pro-rated to t0) minus outputevents (227488 negated); ICU admission (intime) to t0 (NOT the preceding 6 h)	mL/kg	linear; 1 column(s)

Clinical roles and operational details

Covariate	Clinical rationale for adjustment
Age	prognostic; may relate to CRRT prescription and tolerance of fluid removal
Sex	prognostic; body composition/weight normalisation
Race group	Recorded social category; proxy for structural and care-access differences, not a biological mechanism
Admission type	acuity and admission pathway
First ICU type	case mix and unit-level CRRT/charting practice
Calendar period	secular trends in CRRT practice, documentation and outcomes
ICU admission to CRRT start	timing of CRRT initiation; accumulated illness course
CRRT mode	modality may relate to prescribed removal and to severity
Charlson index	baseline comorbidity burden
Chronic kidney disease	CKD alters CRRT indication and fluid tolerance
Heart failure	volume overload/removal targets and prognosis
Chronic liver disease	haemodynamic tolerance and prognosis
Malignancy	prognosis and goals of care
Custom SOFA total	organ dysfunction severity at CRRT start
Documented suspected infection before t0	Documented suspected infection and infection-related treatment; not a validated Sepsis-3 phenotype
Invasive ventilation at t0	respiratory failure; fluid-removal goals
Norepinephrine-equivalent dose	haemodynamic instability limits fluid removal and predicts death
MAP	haemodynamic status
Lactate	hypoperfusion severity
pH	acidaemia as CRRT indication and severity marker
Creatinine	kidney function/indication
BUN	uraemia/indication
Bicarbonate	metabolic acidosis/indication
Potassium	hyperkalaemia/indication
Urine output	residual kidney function and volume status
Cumulative fluid balance	pre-CRRT fluid overload drives removal targets and prognosis

Documented suspected infection required antibiotic and culture events strictly before t0 using the prespecified pairing windows (culture within 24 h after or 72 h before antibiotic initiation). It did not require a Sepsis-3 acute SOFA increment. The custom SOFA used the worst platelet, bilirubin, creatinine, GCS and MAP measurements in [t0 − 24 h, t0). PaO2 values in that window were paired with the nearest eligible pre-t0 FiO2 within 120 min, retaining the lowest paired PaO2–FiO2 ratio; FiO2 candidates extended to t0 − 26 h to permit boundary pairing. The renal component used creatinine only. The cardiovascular component combined minimum MAP with norepinephrine-equivalent dose active at t0; the respiratory component used ventilation status at t0. These implemented component-specific windows differ from the original plan's blanket prior-24-hour description. It is therefore not interchangeable with an unmodified clinical SOFA score. Total SOFA was reconstructed after component imputation. Cumulative fluid balance covers ICU admission to t0, not only the preceding 6 hours; urine output covers the preceding 6 hours and is a total mL/kg quantity. The machine-readable dictionary provides full source IDs, observed missingness, imputation methods and derivation details.

Norepinephrine-equivalent dose (μg/kg/min) = norepinephrine + epinephrine + phenylephrine/10 + dopamine/100 + 2.5 × vasopressin (U/min). The first four rates were expressed in μg/kg/min; phenylephrine recorded in μg/min was divided by analytic weight, norepinephrine in mg/kg/min was multiplied by 1000, and vasopressin in U/h was divided by 60 before conversion. Contributions from infusions active at t0 were summed.

Table S6 Imputation quality control for original scenario runs

ID	Fresh MICE	Continuation	Zero logged events	Structural QC	PSRF <1.2
B	YES	0	TRUE	PASS	TRUE
C	YES	0	TRUE	PASS	TRUE
E	YES	0	TRUE	PASS	TRUE
F	YES	0	TRUE	PASS	TRUE
G	YES	0	TRUE	PASS	TRUE
I	YES	0	TRUE	PASS	TRUE
O	YES	0	TRUE	PASS	TRUE

All seven separate imputation runs used m = 50 and 50 iterations. No continuation or new imputation was performed during revision. Zero logged events, successful structural checks and PSRF below 1.2 describe the recorded diagnostics; they do not prove that the missing-at-random assumption holds. The largest primary final mean PSRF was 1.1773 for the renal SOFA component (Table S9).

Table S7 Missingness by outcome and exposure quartile

Panel A Overall and outcome-stratified missingness

Variable	Overall N = 1453	No recorded 90-day death N = 606	Recorded 90-day death N = 847
CRRT mode	499/1453 (34.3%)	185/606 (30.5%)	314/847 (37.1%)
MAP	2/1453 (0.1%)	Not tabulated	Not tabulated
Lactate	489/1453 (33.7%)	227/606 (37.5%)	262/847 (30.9%)
pH	64/1453 (4.4%)	25/606 (4.1%)	39/847 (4.6%)
Creatinine	13/1453 (0.9%)	8/606 (1.3%)	5/847 (0.6%)
BUN	13/1453 (0.9%)	8/606 (1.3%)	5/847 (0.6%)
Bicarbonate	15/1453 (1.0%)	9/606 (1.5%)	6/847 (0.7%)
Potassium	14/1453 (1.0%)	9/606 (1.5%)	5/847 (0.6%)
Urine output	330/1453 (22.7%)	116/606 (19.1%)	214/847 (25.3%)
Cumulative fluid balance	105/1453 (7.2%)	47/606 (7.8%)	58/847 (6.8%)
SOFA respiratory	260/1453 (17.9%)	117/606 (19.3%)	143/847 (16.9%)
SOFA coagulation	27/1453 (1.9%)	13/606 (2.1%)	14/847 (1.7%)
SOFA hepatic	344/1453 (23.7%)	171/606 (28.2%)	173/847 (20.4%)
SOFA cardiovascular	1/1453 (0.1%)	Not tabulated	Not tabulated
SOFA neurologic	18/1453 (1.2%)	11/606 (1.8%)	7/847 (0.8%)
SOFA renal	13/1453 (0.9%)	8/606 (1.3%)	5/847 (0.6%)
Custom SOFA total	537/1453 (37.0%)	250/606 (41.3%)	287/847 (33.9%)

Panel B Missingness across exposure quartiles

Variable	Q1 N = 364	Q2 N = 363	Q3 N = 363	Q4 N = 363
CRRT mode	141/364 (38.7%)	128/363 (35.3%)	110/363 (30.3%)	120/363 (33.1%)
MAP	Not tabulated	Not tabulated	Not tabulated	Not tabulated
Lactate	115/364 (31.6%)	130/363 (35.8%)	131/363 (36.1%)	113/363 (31.1%)
pH	12/364 (3.3%)	16/363 (4.4%)	18/363 (5.0%)	18/363 (5.0%)
Creatinine	2/364 (0.5%)	1/363 (0.3%)	5/363 (1.4%)	5/363 (1.4%)
BUN	2/364 (0.5%)	1/363 (0.3%)	5/363 (1.4%)	5/363 (1.4%)

Variable	Q1 N = 364	Q2 N = 363	Q3 N = 363	Q4 N = 363
Bicarbonate	3/364 (0.8%)	1/363 (0.3%)	5/363 (1.4%)	6/363 (1.7%)
Potassium	2/364 (0.5%)	1/363 (0.3%)	5/363 (1.4%)	6/363 (1.7%)
Urine output	76/364 (20.9%)	76/363 (20.9%)	89/363 (24.5%)	89/363 (24.5%)
Cumulative fluid balance	26/364 (7.1%)	23/363 (6.3%)	26/363 (7.2%)	30/363 (8.3%)
SOFA respiratory	65/364 (17.9%)	71/363 (19.6%)	68/363 (18.7%)	56/363 (15.4%)
SOFA coagulation	6/364 (1.6%)	3/363 (0.8%)	9/363 (2.5%)	9/363 (2.5%)
SOFA hepatic	78/364 (21.4%)	79/363 (21.8%)	101/363 (27.8%)	86/363 (23.7%)
SOFA cardiovascular	Not tabulated	Not tabulated	Not tabulated	Not tabulated
SOFA neurologic	3/364 (0.8%)	5/363 (1.4%)	4/363 (1.1%)	6/363 (1.7%)
SOFA renal	2/364 (0.5%)	1/363 (0.3%)	5/363 (1.4%)	5/363 (1.4%)
Custom SOFA total	128/364 (35.2%)	139/363 (38.3%)	143/363 (39.4%)	127/363 (35.0%)

Values are missing n/denominator (%), before imputation. Exact overall counts are reported. Outcome- and quartile-specific breakdowns were not tabulated for MAP and the cardiovascular SOFA component, which have only two and one missing values, respectively. The total SOFA row describes missingness of the constructed score, not an additional imputation input. Quartile boundaries derive from the final cohort distribution; fixed model contrast values derive from the pre-outcome distribution. Using final-cohort quartiles q25, q50 and q75, groups were Q1 ≤ q25, Q2 > q25 to ≤ q50, Q3 > q50 to ≤ q75, and Q4 > q75.

Missing imputation inputs per patient

Number of missing imputation inputs	Patients
0	361
1	472
2	333
3	178
4	68
>=5	41

This count refers to the 16 imputation inputs before imputation, with the six SOFA components counted separately and the reconstructed SOFA total not counted. It gives 472 patients with exactly one missing imputation input. When total SOFA is instead counted as a single model input, 490 patients have exactly one missing model input (Table S11, Panel B); the 361 complete cases and 1092 excluded patients are the same under both definitions. The full multidimensional pattern table is not expanded into small individual patterns.

Table S8 Primary imputation methods and predictor roles

Imputed variable	Method	Missing n	Predictors	Visit order
CRRT mode	logreg	499	36	13
MAP	pmm	2	36	22
Lactate	pmm	489	36	23
pH	pmm	64	36	24
Creatinine	pmm	13	36	25
BUN	pmm	13	36	26
Bicarbonate	pmm	15	36	27
Potassium	pmm	14	36	28
Urine output	pmm	330	36	29
Cumulative fluid balance	pmm	105	36	30
SOFA respiratory	polr	260	36	32
SOFA coagulation	polr	27	36	33
SOFA hepatic	polr	344	36	34
SOFA cardiovascular	polyreg	1	36	35
SOFA neurologic	polr	18	36	36
SOFA renal	polr	13	36	37

Predictor-only fields comprised 90-day mortality, event time, all three exposure spline basis terms, complete adjustment variables and the number of observed SOFA components. These variables were not imputed. The 37-by-37 predictor matrix is supplied as an accompanying TSV, and the full methods/roles TSV includes every variable. All imputed inputs had 36 predictors in the stored matrix. Visit order follows the full variable sequence, so the displayed imputed rows are not consecutively numbered.

Imputation fact	Value
Completed datasets	50
Iterations	50
Seed	42
Logged events	0
Missing outcomes or exposure imputed	No
Total SOFA included as an imputation target	No; reconstructed from six components afterward

Imputation fact	Value
Stored chain mean/variance dimensions	37 variables × 50 iterations × 50 chains

Table S9 Observed and imputed distributions and convergence

Observed distributions describe available values. Imputed distributions describe only originally missing cells, summarized within each of the 50 completed datasets and then by the median and range across datasets. Differences are expected when missingness is associated with observed covariates and outcomes; these comparisons do not validate the missing-at-random assumption.

Lactate

Category or summary	Observed n	Observed % or median	Imputed cells	Imputed % or median across 50	Range across 50
<2	409	42.4	489	57	49.7–65.4
2-<4	257	26.7	489	27.6	22.7–32.9
4-<8	142	14.7	489	10.8	7.4–15.5
>=8	156	16.2	489	4.2	2.5–6.1
median	964	2.3	489	1.8	1.6–2

Median rows use the variable's original unit; categorical rows use percentages. Observed n in category rows is the category count; n in the median row is the observed denominator.

Urine output

Category or summary	Observed n	Observed % or median	Imputed cells	Imputed % or median across 50	Range across 50
<1	645	57.4	330	64.2	57.3–70.6
1-<3	268	23.9	330	20.9	15.2–25.8
3-<6	119	10.6	330	9.2	5.5–13
>=6	91	8.1	330	6.1	3.3–8.8
median	1123	0.68	330	0.508	0.4–0.658

Median rows use the variable's original unit; categorical rows use percentages. Observed n in category rows is the category count; n in the median row is the observed denominator.

pH

Category or summary	Observed n	Observed % or median	Imputed cells	Imputed % or median across 50	Range across 50
<7.20	186	13.4	64	6.2	1.6–14.1
7.20-<7.30	401	28.9	64	26.6	17.2–39.1

Category or summary	Observed n	Observed % or median	Imputed cells	Imputed % or median across 50	Range across 50
7.30-<7.40	548	39.5	64	43.8	31.2–51.6
>=7.40	254	18.3	64	23.4	14.1–32.8
median	1389	7.32	64	7.338	7.31–7.36

Median rows use the variable’s original unit; categorical rows use percentages. Observed n in category rows is the category count; n in the median row is the observed denominator.

Cumulative fluid balance

Category or summary	Observed n	Observed % or median	Imputed cells	Imputed % or median across 50	Range across 50
<0	85	6.3	105	12.4	5.7–21.9
0-<25	225	16.7	105	30.5	18.1–41
25-<50	173	12.8	105	16.2	7.6–23.8
50-<100	263	19.5	105	16.7	11.4–23.8
>=100	602	44.7	105	23.8	15.2–35.2
median	1348	84.409	105	33.69	20.349–49.07

Median rows use the variable’s original unit; categorical rows use percentages. Observed n in category rows is the category count; n in the median row is the observed denominator.

Custom SOFA total

Category or summary	Observed n	Observed % or median	Imputed cells	Imputed % or median across 50	Range across 50
0-9	29	3.2	537	25.5	22.7–28.3
10-12	119	13	537	26.8	22.9–30
13-15	315	34.4	537	26.2	23.1–29.4
16-18	331	36.1	537	16.4	14.3–19.7
19-24	122	13.3	537	4.7	3.5–6.9
median	916	15	537	12	12–12

Median rows use the variable’s original unit; categorical rows use percentages. Observed n in category rows is the category count; n in the median row is the observed denominator.

CRRT mode

Category or summary	Observed n	Observed % or median	Imputed cells	Imputed % or median across 50	Range across 50
CVVHDF	901	94.4	499	89	84.6–92.2
OTHER_KNOWN	53	5.6	499	11	7.8–15.4

SOFA respiratory

Category or summary	Observed n	Observed % or median	Imputed cells	Imputed % or median across 50	Range across 50
0	32	2.7	260	27.7	22.3–33.8
1	52	4.4	260	18.8	13.8–25.4
2	250	21	260	33.5	26.9–41.2
3	405	33.9	260	15	10.4–20
4	454	38.1	260	4.6	2.3–8.8

SOFA coagulation

Category or summary	Observed n	Observed % or median	Imputed cells	Imputed % or median across 50	Range across 50
0	561	39.3	27	59.3	37–81.5
1	264	18.5	27	14.8	3.7–29.6
2	361	25.3	27	14.8	3.7–37
3	200	14	27	7.4	0–22.2
4	40	2.8	27	0	0–7.4

SOFA hepatic

Category or summary	Observed n	Observed % or median	Imputed cells	Imputed % or median across 50	Range across 50
0	412	37.2	344	38.7	34.9–45.6
1	148	13.3	344	15.4	11.3–18.9
2	273	24.6	344	24.7	18.3–29.1
3	110	9.9	344	9.6	6.1–11.9
4	166	15	344	11.6	8.1–16

SOFA neurologic

Category or summary	Observed n	Observed % or median	Imputed cells	Imputed % or median across 50	Range across 50
0	142	9.9	18	16.7	5.6–27.8
1	133	9.3	18	11.1	0–27.8
2	109	7.6	18	5.6	0–22.2
3	316	22	18	16.7	0–38.9
4	735	51.2	18	50	27.8–66.7

SOFA renal

Category or summary	Observed n	Observed % or median	Imputed cells	Imputed % or median across 50	Range across 50
0	15	1	13	0	0–7.7
1	79	5.5	13	0	0–7.7
2	408	28.3	13	7.7	0–23.1
3	438	30.4	13	15.4	0–46.2
4	500	34.7	13	69.2	53.8–92.3

Final mean PSRF summaries

Variable	Final mean PSRF
CRRT mode	1.0027
MAP	1.0053
Lactate	0.9996
pH	0.9985
Creatinine	1.0417
BUN	1.021
Bicarbonate	1.0085
Potassium	1.0015
Urine output	1.0012
Cumulative fluid balance	0.9956
SOFA respiratory	1.0013
SOFA coagulation	1.0024

Variable	Final mean PSRF
SOFA hepatic	1.0017
SOFA cardiovascular	1.006
SOFA neurologic	1.0084
SOFA renal	1.1773

Final mean PSRF values ranged from 0.9956 to 1.1773; renal SOFA had the largest value. The prespecified threshold was 1.2, but this threshold does not demonstrate perfect chain mixing or correct imputation assumptions. Figure S4 displays traces for the eight inputs with at least 27 originally missing cells: CRRT mode, lactate, pH, urine output, cumulative fluid balance, and respiratory, coagulation and hepatic SOFA. Traces are not displayed for neurologic SOFA (18 cells), renal SOFA (13 cells; PSRF 1.1773), cardiovascular SOFA, MAP, creatinine, BUN, bicarbonate or potassium. Exact counts, methods and final PSRF are retained for all imputed inputs. Distribution reporting used a separate selection: cell-value distributions were not supplied for cardiovascular SOFA, MAP, creatinine, BUN, bicarbonate and potassium, each with at most 15 missing cells; neurologic and renal SOFA distributions are supplied as categorical summaries.

Table S10 Primary model diagnostics

Panel A Apparent calibration

Metric	Median	Minimum	Maximum
Brier score (apparent)	0.1983	0.1955	0.2001
mean predicted minus observed (calibration-in-the-large)	0.0000	0.0000	0.0000
calibration intercept (offset logistic; apparent)	0.0000	0.0000	0.0000
calibration slope (apparent)	1.0000	1.0000	1.0000

These are apparent, training-data diagnostics, not external or optimism-corrected predictive validation. Calibration-in-the-large and intercept near zero, and slope near one, are expected from fitting and evaluating an intercept-containing maximum-likelihood logistic model on the same data. The median Brier score was 0.1983 (range 0.1955–0.2001). Calibration is secondary to the association estimand.

Panel B Influence on exposure contrasts

Metric within each completed dataset	Median across 50	Minimum	Maximum
Q25 absolute approximate change / SE: patient P95	0.0540	0.0533	0.0550
Q25 absolute approximate change / SE: patient P99	0.0636	0.0620	0.0657
Q25 absolute approximate change / SE: patient maximum	0.3457	0.1943	0.5028
Q25 patients with absolute change / SE >1	0	0	0
Q75 absolute approximate change / SE: patient P95	0.0551	0.0538	0.0560
Q75 absolute approximate change / SE: patient P99	0.0673	0.0652	0.0691

Metric within each completed dataset	Median across 50	Minimum	Maximum
Q75 absolute approximate change / SE: patient maximum	0.1822	0.1057	0.2663
Q75 patients with absolute change / SE >1	0	0	0
Cook distance patient P99	0.0039	0.0037	0.0042
Cook distance patient maximum	0.0093	0.0077	0.0215
Patients with Cook distance >4/N	41	37	48
Leverage patient P99	0.0777	0.0754	0.0795
Leverage patient maximum	0.6208	0.4923	0.6941

Influence summaries use case-deletion approximations from fitted models, not refits after excluding selected observations. No observation was removed based on these diagnostics. The median count above the descriptive Cook-distance threshold was 41; this threshold is not an exclusion rule.

Panel C Design matrix collinearity

Term group	df	Generalized index median	Adjusted index median	Adjusted index maximum
Charlson index	1	2.755	1.660	1.668
Custom SOFA total	1	2.142	1.464	1.484
Chronic kidney disease	1	2.103	1.450	1.454
Bicarbonate	1	1.934	1.391	1.407
pH	1	1.910	1.382	1.392
Lactate	1	1.887	1.374	1.397
Chronic liver disease	1	1.868	1.367	1.372
Invasive ventilation at t0	1	1.802	1.343	1.354
Norepinephrine-equivalent dose	1	1.792	1.339	1.346
Malignancy	1	1.781	1.334	1.338
Heart failure	1	1.680	1.296	1.300
Cumulative fluid balance	1	1.644	1.282	1.294
ICU admission to CRRT start	1	1.627	1.275	1.281
Creatinine	1	1.599	1.264	1.277
BUN	1	1.594	1.263	1.270
Age	1	1.390	1.179	1.183

Term group	df	Generalized index median	Adjusted index median	Adjusted index maximum
Potassium	1	1.244	1.115	1.121
Documented suspected infection before t0	1	1.230	1.109	1.116
MAP	1	1.211	1.101	1.107
Urine output	1	1.202	1.096	1.107
First ICU type	6	2.913	1.093	1.095
Sex	1	1.140	1.068	1.073
Admission type	5	1.677	1.053	1.055
Calendar period	4	1.445	1.047	1.049
Race group	4	1.394	1.042	1.044
CRRT mode	1	1.077	1.038	1.053
Exposure spline	3	1.220	1.034	1.035

The determinant-based generalized collinearity index was computed from the unweighted correlation matrix of the design columns, grouping multi-column terms; the adjusted index is the 1/(2 × df) power. It describes design-matrix collinearity and is not a coefficient-covariance-based generalized VIF for the fitted logistic model. The maximum adjusted index across imputations was 1.668. All 50 primary full-model fits converged with full rank, finite coefficients and identical coefficient naming.

Table S11 Complete-case and covariate-spline sensitivity details

Panel A Complete-case population and fit

Quantity	Value
N complete cases	361
events (90-day deaths)	208
non-events	153
excluded (>=1 missing model input)	1092
events among excluded	639
non-events among excluded	453

The complete-case analysis retained all primary adjustment terms and fixed exposure knots/contrasts. Its design matrix had 45 columns including the intercept and rank 45. Full, linear-exposure and no-exposure models converged using ordinary maximum likelihood, with finite coefficients and no glm warnings. Estimates were stable when iteration limits were increased from 25 to 100 and 1000. All categorical levels contained both outcome classes. These checks did not constitute a formal separation test; no Firth fallback

was used. The largest absolute coefficient was the intercept, 38.95 (SE 14.50), consistent with the uncentred pH term, rather than a reason to remove a variable. This sensitivity was declared in SAP Section 10 and was reassessed and reported in detail during revision.

The complete-case exposure median was 3.8122 mL/kg/h (Q1 2.7335; Q3 5.1480); 89 patients were below the fixed Q25 contrast, 174 between fixed Q25 and Q75, and 98 above Q75. All fixed knots and contrast values were inside the observed complete-case range. Results, including standardized risks over the complete-case population, appear in Table S2. Complete cases represent a selected subset, and their wider confidence intervals should not be interpreted as equivalence to the imputed results.

Panel B Missing inputs contributing to complete-case exclusion

Variable	Missing among 1092 excluded	Only missing model input
CRRT mode	499	184
Custom SOFA total	537	131
MAP	2	0
Lactate	489	112
pH	64	1
Creatinine	13	0
BUN	13	0
Bicarbonate	15	0
Potassium	14	0
Urine output	330	62
Cumulative fluid balance	105	0

Rows overlap when a patient has multiple missing inputs. These summaries describe why patients were not complete cases; they are not a complete baseline comparison between complete and incomplete cases.

Panel C Joint nonlinear covariate sensitivity

During revision, a single sensitivity model jointly represented age, lactate, norepinephrine-equivalent dose, cumulative ICU-admission-to-t0 fluid balance and 6-hour urine output with three-knot restricted cubic splines. Knots were fixed at their observed 10th, 50th and 90th percentiles before fitting this sensitivity. The exposure spline, remaining covariates and stored primary imputations were unchanged. Imputations were not regenerated to include the added nonlinear covariate terms; this limits claims of full compatibility between the sensitivity model and the original imputation models. Full, no-exposure and linear-exposure versions produced 150 converged, full-rank fits with finite estimates; the full model had 50 columns including the intercept.

Variable	Observed n	P10 knot	P50 knot	P90 knot
Age	1453	42.0000	64.0000	81.0000
Lactate	964	1.0000	2.3000	10.7700
Norepinephrine-equivalent dose	1453	0.0000	0.1003	0.5522
Cumulative fluid balance	1348	4.0852	84.4086	267.9819
Urine output	1123	0.0457	0.6796	5.3252

The overall exposure test was P = 0.479 and the nonlinearity test P = 0.524. Q75 versus reference yielded OR 1.056 (95% CI 0.872–1.280) and RD +1.1 percentage points (−2.7 to +4.8). These results were close to the primary estimates. All contrasts are reported in Table S2; no variable was selected or removed according to this result.

Table S12 Exposure processing and distribution

Panel A Record retention and quality flags

Processing step or overlapping descriptor	Records	Patients
all 224191 records in the index ICU stay (any time)	189675	1453
charttime in [t0, t0+12h)	18049	1453
numeric value present (valuenum not null)	18049	1453
unit = mL	18049	1453
inside a raw active CRRT interval	18032	1453
excluded at step 5: in window but outside active CRRT interval	17	16
duplicate records: same charttime, superseded by a later-stored record (not retained)	0	0
charttimes with >1 record at step 5 (duplicate charttimes)	0	0
unresolved ambiguous charttimes: latest storetime tied with discordant values (charttime dropped)	0	0
retained records; one per charttime	18032	1453
retained records with negative value (retained in primary)	51	14
retained records with value = 0 (retained in primary)	778	406
retained records with warning = 1 (retained in primary)	353	166
records exactly at t0 (included)	710	710
records exactly at L = t0+12h (excluded by the half-open window; used by sensitivity B)	295	295

The sequential retention steps and overlapping flags are not additive. Patient denominator is 1453. All retained records had numeric values and mL units. Negative and warning-flagged records remained in the primary definition and were separately excluded in sensitivities F and G.

Panel B Patient and record distributions

Measure	Observed n	Median [Q1–Q3]	P1–P99
Admission weight kg	1315	87.60 [73.00–103.00]	48.43–171.49
Analytic weight kg	1453	87.50 [73.00–103.00]	47.81–173.23
Distinct charttimes per represented bin	1453	1.08 [1.00–1.09]	1.00–1.36
Patient mean recorded volume mL	1453	341.67 [247.69–426.15]	36.95–687.01
Patient exposure mL/kg/h	1453	3.79 [2.73–5.02]	0.43–9.13
Charttimes beyond represented bins	1453	1.00 [0.00–1.00]	0.00–4.00
Represented bins first 6 h	1453	6.00 [6.00–6.00]	4.00–6.00
Represented bins second 6 h	1453	6.00 [6.00–6.00]	4.00–6.00
Retained charttimes per patient	1453	12.00 [12.00–13.00]	10.00–16.00
Represented bins	1453	12.00 [11.00–12.00]	10.00–12.00

Across the 18032 retained records, the recorded-volume median was 350 mL [Q1–Q3 220–450]; P1–P99 0–800 mL. The patient-exposure range was −0.35 to 55.74 mL/kg/h. No winsorization or tail deletion was applied; the displayed fitted curve is restricted to the prespecified pre-outcome P1–P99 support. The display limits are 0.4197–9.1308 mL/kg/h from the pre-outcome N = 1465 distribution, whereas this panel describes the final N = 1453 cohort.

Panel C Recording coverage and CRRT duration

Descriptor	Count category	Patients
Bins first 6 h	4	51
Bins first 6 h	5	232
Bins first 6 h	6	1170
Bins second 6 h	4	63
Bins second 6 h	5	235
Bins second 6 h	6	1155
Retained charttimes	10	58
Retained charttimes	11	133
Retained charttimes	12	608
Retained charttimes	13	529

Descriptor	Count category	Patients
Retained charttimes	14	93
Retained charttimes	15	16
Retained charttimes	>=16	16
Represented bins	10	194
Represented bins	11	307
Represented bins	12	952

All 1453 analytic weights came from procedure-event patientweight. Admission weight was separately available for 1315 patients. More charttimes than represented bins occurred in 873 patients, explaining why record weighting is not identical to bin weighting. There were 1406 patients with a full 12 documented active CRRT hours and 47 with interrupted active intervals during the window; the 1st percentile of active treatment time was 10.5 h. Later CRRT rows occurred in more than one ICU stay for 114 patients, but only the selected index stay contributed to the exposure. The retained measurement count ranged from 10 to 20 per patient. The weight hierarchy was valid procedure patientweight, then the closest preceding daily weight (item 224639) within the index stay during [t0 − 7 days, t0), then preceding admission weight (item 226512); valid weights were 20–300 kg. The daily-weight fallback did not determine exposure in the final cohort.

Figure S1 Exposure distribution and standardized risk difference

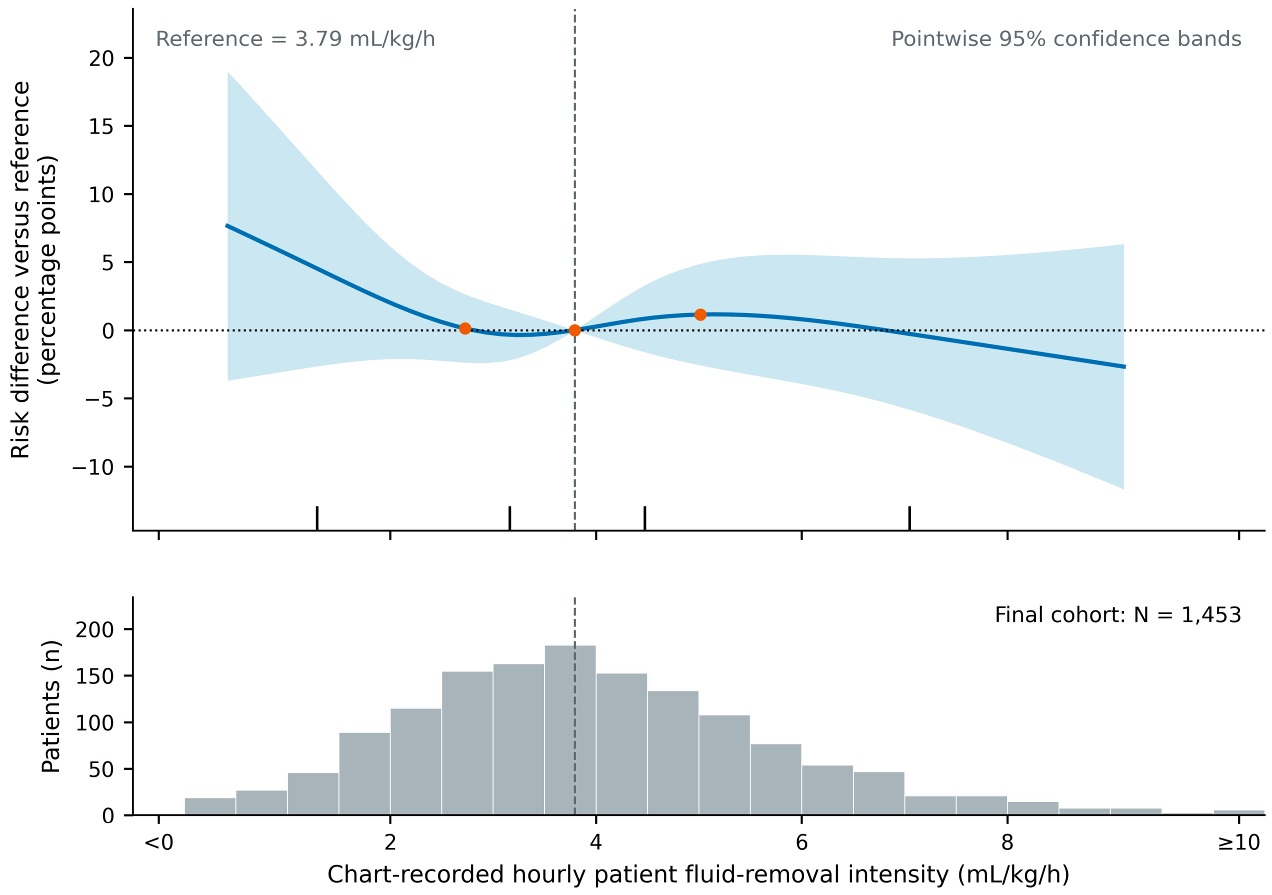

Curve display: 0.42–9.13 mL/kg/h; short ticks mark spline knots.
Histogram: 0.5-unit bins with open-ended tail categories; all patients included.

The curve shows the adjusted 90-day risk difference from the fixed reference 3.7932 mL/kg/h with pointwise 95% confidence bands. Counts describe the N = 1453 analytic cohort; curve support is the fixed pre-outcome P1–P99 range, 0.4197–9.1308 mL/kg/h (N = 1465). The histogram includes all patients, using open-ended categories below 0 and at or above 10 mL/kg/h.

Figure S2 Patient summaries of charted fluid-removal fields

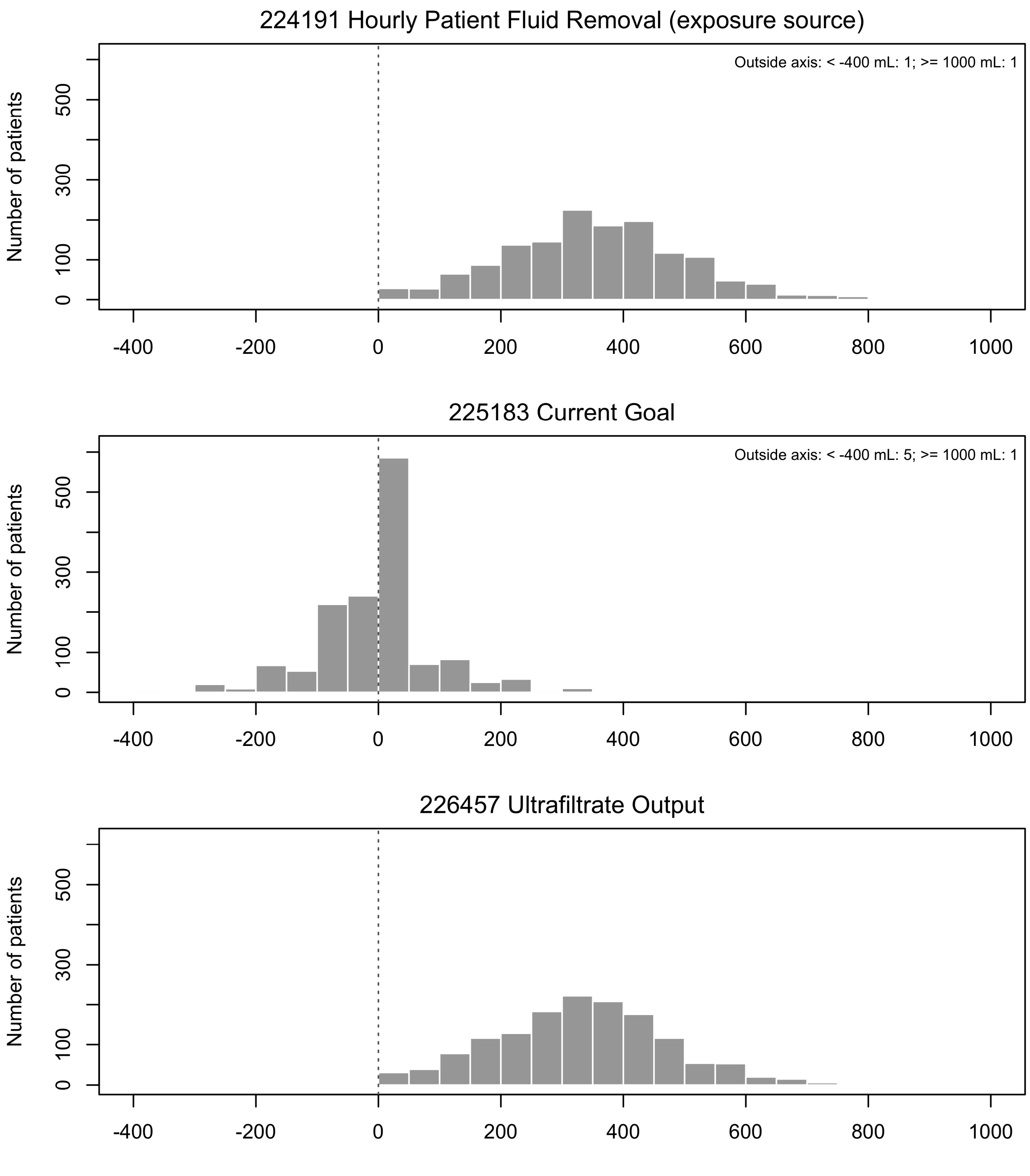

Each panel summarizes per-patient median recorded values within the same early active-CRRT window. Units are mL as recorded; comparator measurement intervals are undocumented. The figure is descriptive and does not validate equivalence to achieved UFNET.

Figure S3 Absolute risk differences across analyses

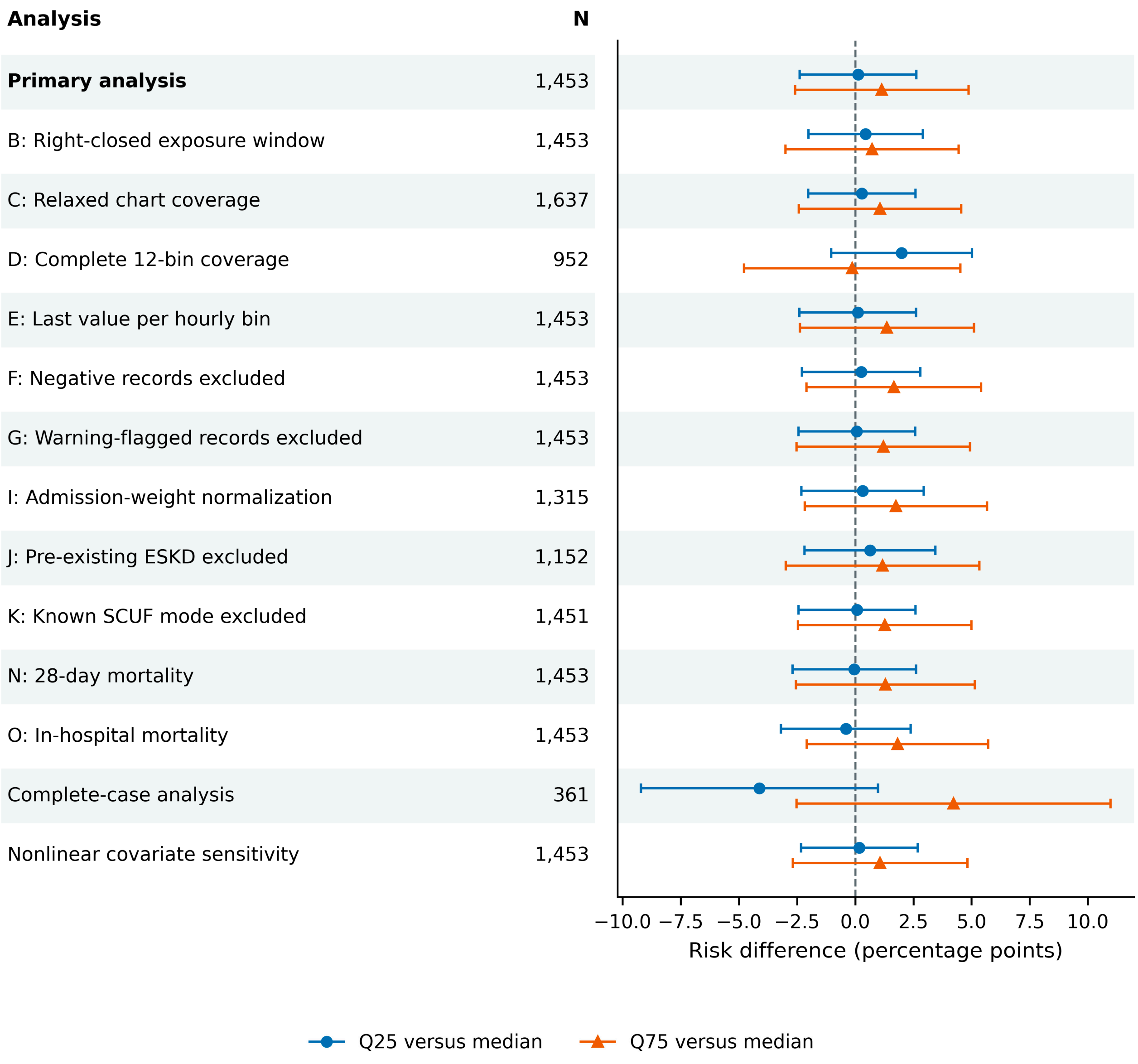

Pointwise 95% confidence intervals; overlapping analyses are not independent replications.

Q25 and Q75 contrasts use the same fixed exposure values. Horizontal intervals are pointwise 95% confidence intervals; estimates are risk differences in percentage points. The complete-case sensitivity was declared in SAP Section 10 and was reassessed and reported in detail during revision; the covariate-spline sensitivity was specified at revision. Analysis populations and event counts are reported in Table S2. Analyses overlap and are not independent replications.

Figure S4 Multiple imputation chain traces

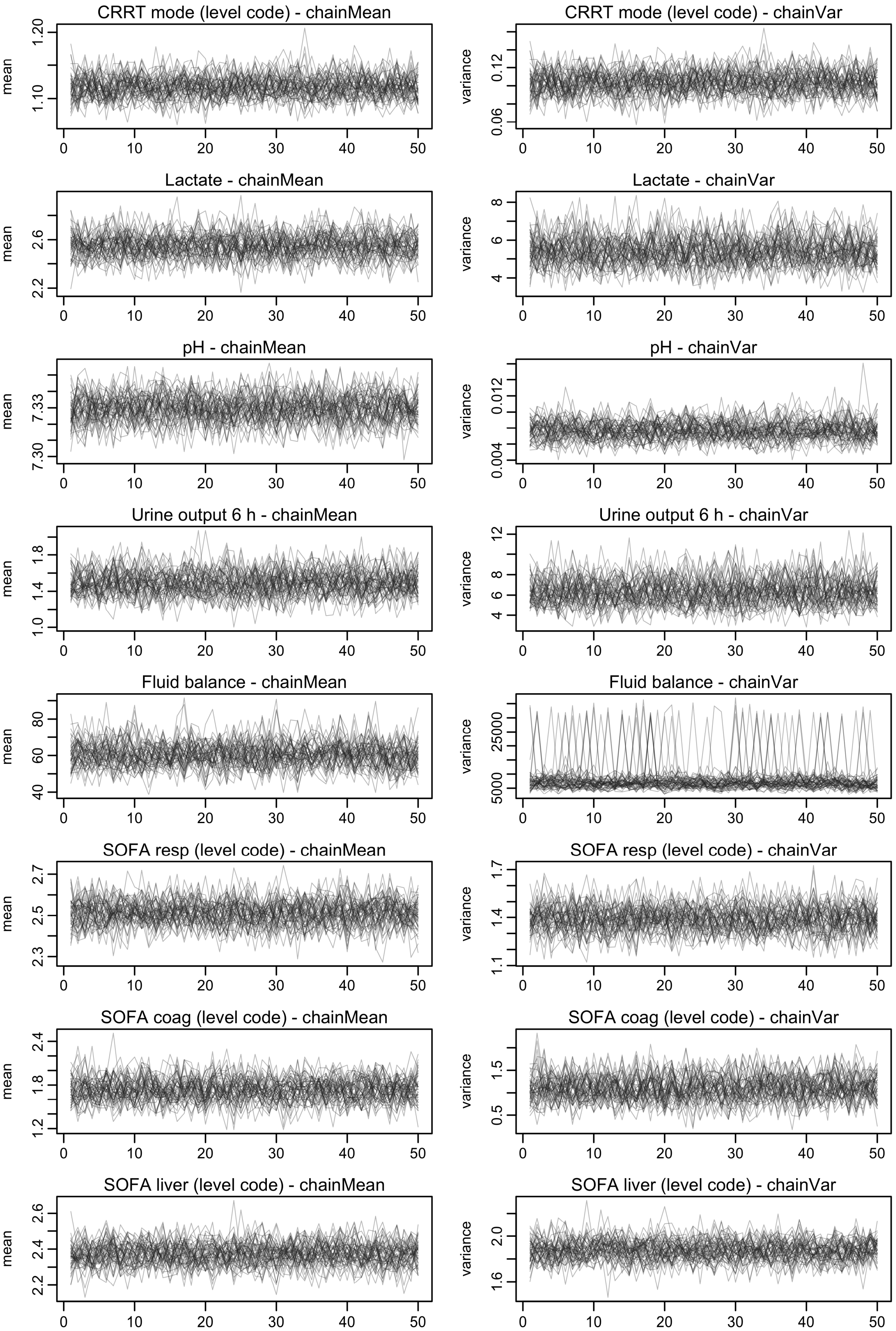

Stored chain-mean and chain-variance traces from the primary 50-imputation, 50-iteration run are shown for the eight inputs with at least 27 originally missing cells. The lines are imputation diagnostics, not patient trajectories. Table S9 identifies all eight inputs without displayed traces, including renal SOFA (13 missing cells; final mean PSRF 1.1773), and separately describes the available cell-value distributions.

Figure S5 Apparent calibration

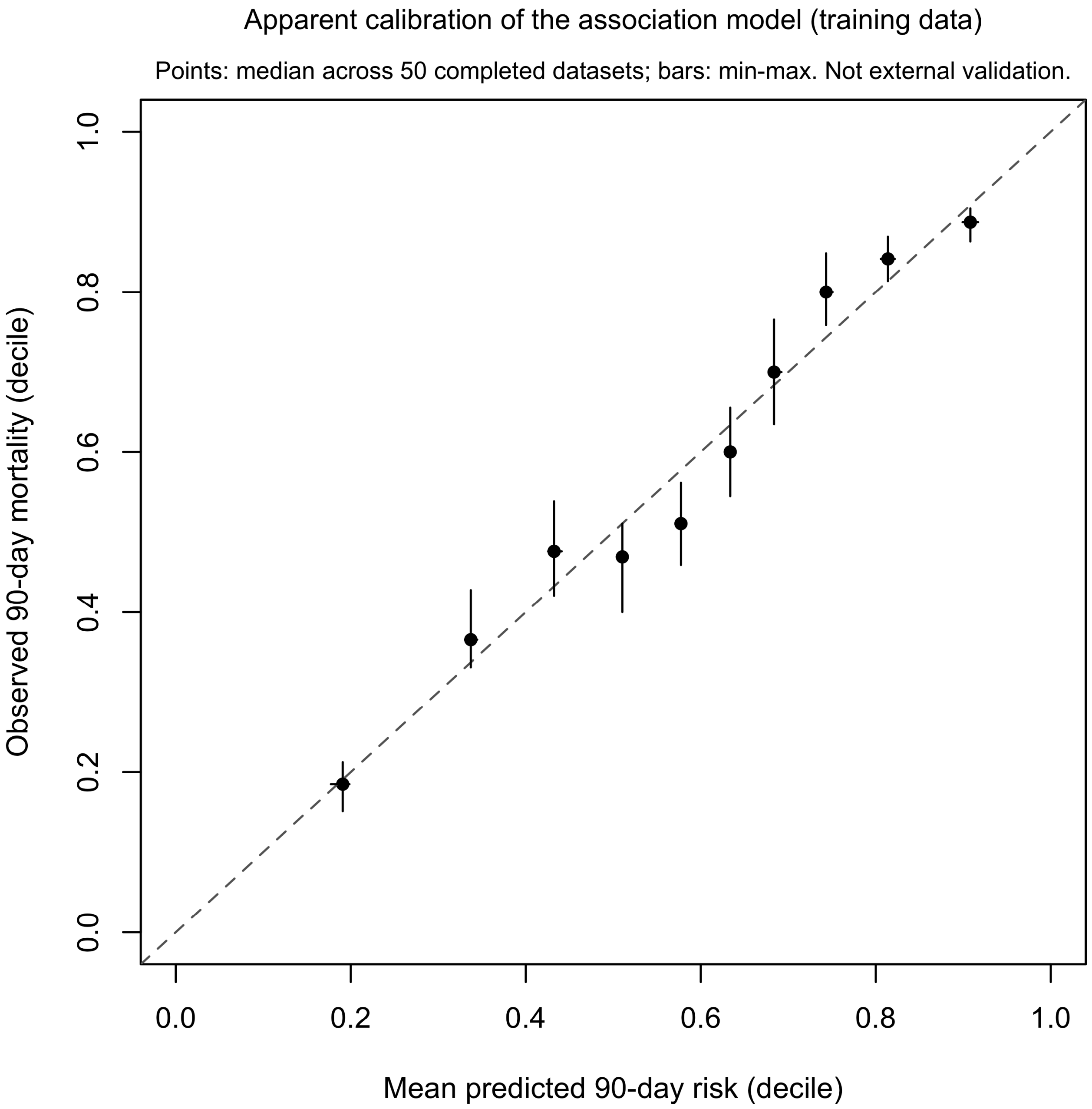

Decile points show median observed and predicted 90-day risks across completed datasets; bars show the range across imputations. Evaluation uses the model-fitting data and is not external or optimism-corrected validation.
